# An improved ENSO simulation by representing chlorophyll-induced climate feedback in the NCAR Community Earth System Model

**DOI:** 10.1038/s41598-017-17390-2

**Published:** 2017-12-07

**Authors:** Xianbiao Kang, Rong-Hua Zhang, Chuan Gao, Jieshun Zhu

**Affiliations:** 1 Civil Aviation Flight University of China, Guanghan, 618307 China; 2 Key Laboratory of Ocean Circulation and Waves, Institute of Oceanology, Chinese Academy of Sciences, Qingdao, 266071 China; 3 Laboratory for Ocean and Climate Dynamics, Qingdao National Laboratory for Marine Science and Technology, Qingdao, 266237 China; 4 University of Chinese Academy of Sciences, Beijing, China; 5 Earth System Science Interdisciplinary Center (ESSIC), University of Maryland, College Park, MD 20740 Maryland USA

## Abstract

The El Niño-Southern oscillation (ENSO) simulated in the Community Earth System Model of the National Center for Atmospheric Research (NCAR CESM) is much stronger than in reality. Here, satellite data are used to derive a statistical relationship between interannual variations in oceanic chlorophyll (CHL) and sea surface temperature (SST), which is then incorporated into the CESM to represent oceanic chlorophyll -induced climate feedback in the tropical Pacific. Numerical runs with and without the feedback (referred to as feedback and non-feedback runs) are performed and compared with each other. The ENSO amplitude simulated in the feedback run is more accurate than that in the non-feedback run; quantitatively, the Niño3 SST index is reduced by 35% when the feedback is included. The underlying processes are analyzed and the results show that interannual CHL anomalies exert a systematic modulating effect on the solar radiation penetrating into the subsurface layers, which induces differential heating in the upper ocean that affects vertical mixing and thus SST. The statistical modeling approach proposed in this work offers an effective and economical way for improving climate simulations.

## Introduction

Substantial biases still exist in climate models used to simulate the El Niño-Southern oscillation (ENSO) in the tropical Pacific^1,2^, which is the strongest interannual signals in the climate system^3–6^. Recent studies indicate that interannual chlorophyll (CHL) anomalies (CHLAs) can have significant modulating effects on ENSO in the tropical Pacific^1,7–20^. However, large uncertainties exist in the way the CHL-induced feedback is represented in climate simulations, and this affects the models used to depict ENSO in the tropical Pacific^20^. In the current NCAR CESM, for instance, oceanic biology-induced heating effects may not be adequately represented^1^. Although the ocean model itself in the CESM includes a biogeochemical component that can be used to describe interactions between the biological and physical systems in the ocean, the model is not accurate enough for its practical use and must be improved adequately^1^. Additionally, the CESM with an interactive biogeochemical component is extremely time-consuming and thus impractical to run extensively. Thus, CHL is commonly prescribed as seasonally varying climatology derived from satellite data. As such, interannually varying effects of oceanic biology-induced heating on the physical system are not adequately taken into account. As a result, the CESM without an interannually varying bio-feedback tends to simulate a stronger ENSO than in reality. As the NCAR CESM is widely used by scientists all over the world^21,22^, an effective way to reduce biases in its ENSO simulations is urgently needed.

**Figure 1:**
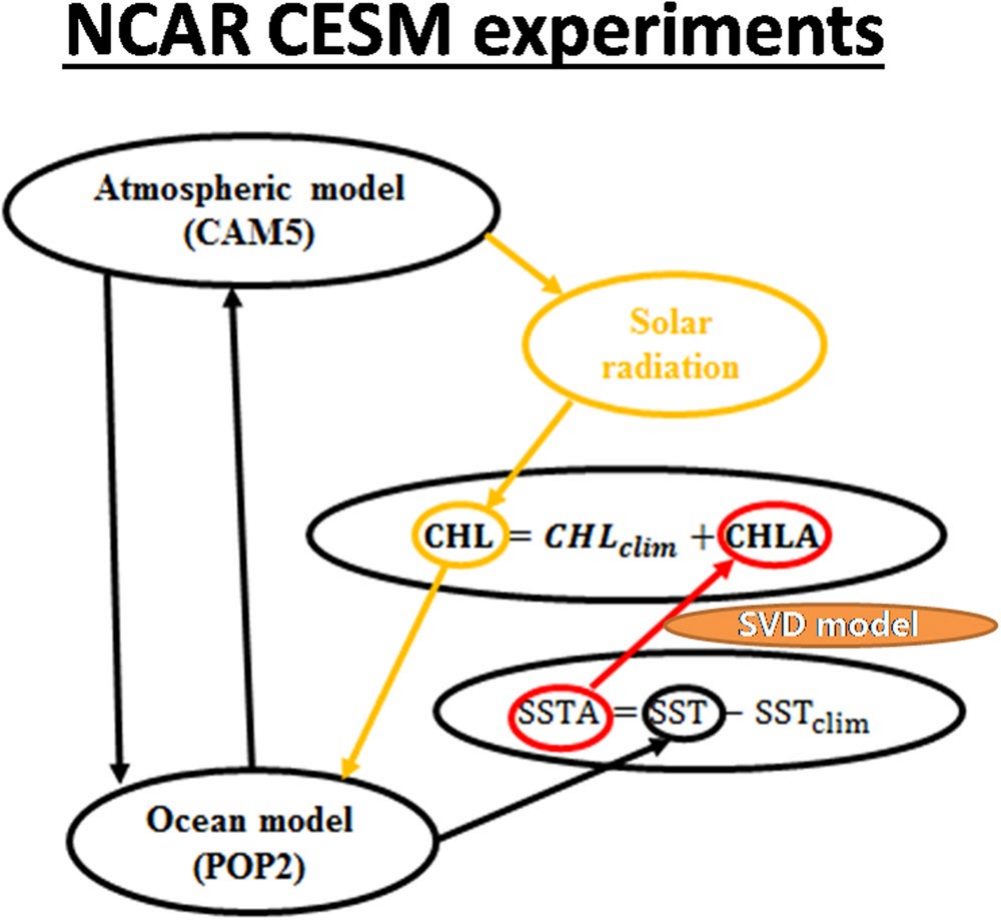
A schematic diagram illustrating the inclusion of the CHL-induced bio-feedback using the NCAR CESM, consisting of the original atmosphere model (CAM5) and the OGCM (POP2), and an embedded statistical representation for interannual CHL variability developed in this work. The total CHL field is separated into its climatological component (CHL_clim_) and interannual anomaly component (CHL_inter_), written as CHL = CHL_clim_ + CHL_inter_, which directly affect the vertical distribution of the incoming solar radiation in the upper ocean. The statistical relationships between the interannual variations in SST and CHL are derived from satellite observations and used to explicitly calculate CHLAs in the CESM to represent CHL-induced heating effects.

Here, we aim to improve ENSO simulations in the CESM by considering the oceanic biology-induced heating effects^17^. As more satellite data become available, CHL variations are used to represent oceanic biology-induced climate feedback effects^23^. In the tropical Pacific, oceanic physical changes are the dominant driving force that produces interannual CHL anomalies (CHLAs), while oceanic biological fields represent a response^19^. Based on satellite data, a close relationship exists between the interannual variations in CHL and SST, which provide a physical basis to derive CHLAs from SSTAs. A statistical model for interannual CHL variations can be derived from satellite data (the details are given in the Supplementary section); the derived CHLA model is then implemented into the NCAR CESM to represent oceanic biology-induced feedbacks (Fig. 1). Furthermore, the NCAR CESM-based experiments are conducted to examine the bio-effect on and the underlying processes of ENSO simulations (Table 1 and see Methods in details).

**Table 1:** Experiments performed to demonstrate the CHL-induced heating effects using the CESM. CHL_clim_ is a climatological run that uses CHL as its seasonally varying climatology prescribed from satellite observations. CHL_inter_ is an interannual run that considers the interannually varying CHL effects (interannual CHL anomalies calculated from the SST anomalies by using the statistical CHL model). In addition, an El Niño case in the model years 153 and 154 is selected for two more experiments that use the CESM designed to illustrate direct effects of CHLAs on SST differences and the underlying processes responsible for the differences in two runs; CHL_clim_–EN is a run that uses CHL as its seasonally varying climatology, and CHL_inter_–EN is a run that considers the interannually varying CHL effects. These two runs are restarted from the same initial state at model year 153 and are time integrated for 2 years. The results from these two runs are compared with each other to identify the effects on the detailed evolution of some related fields.

Experiment names	How CHL is represented	Time integration performed
CHL_clim_	Climatological CHL	Years 151–200
CHL_inter_	Climatological CHL + CHLA from the statistical model	Years 151–200
CHL_clim_-EN	Climatological CHL	El Niño case for years 153–154
CHL_inter_-EN	Climatological CHL + CHLA from the statistical model	El Niño case for years 153–154

## Results

**Figure 2:**
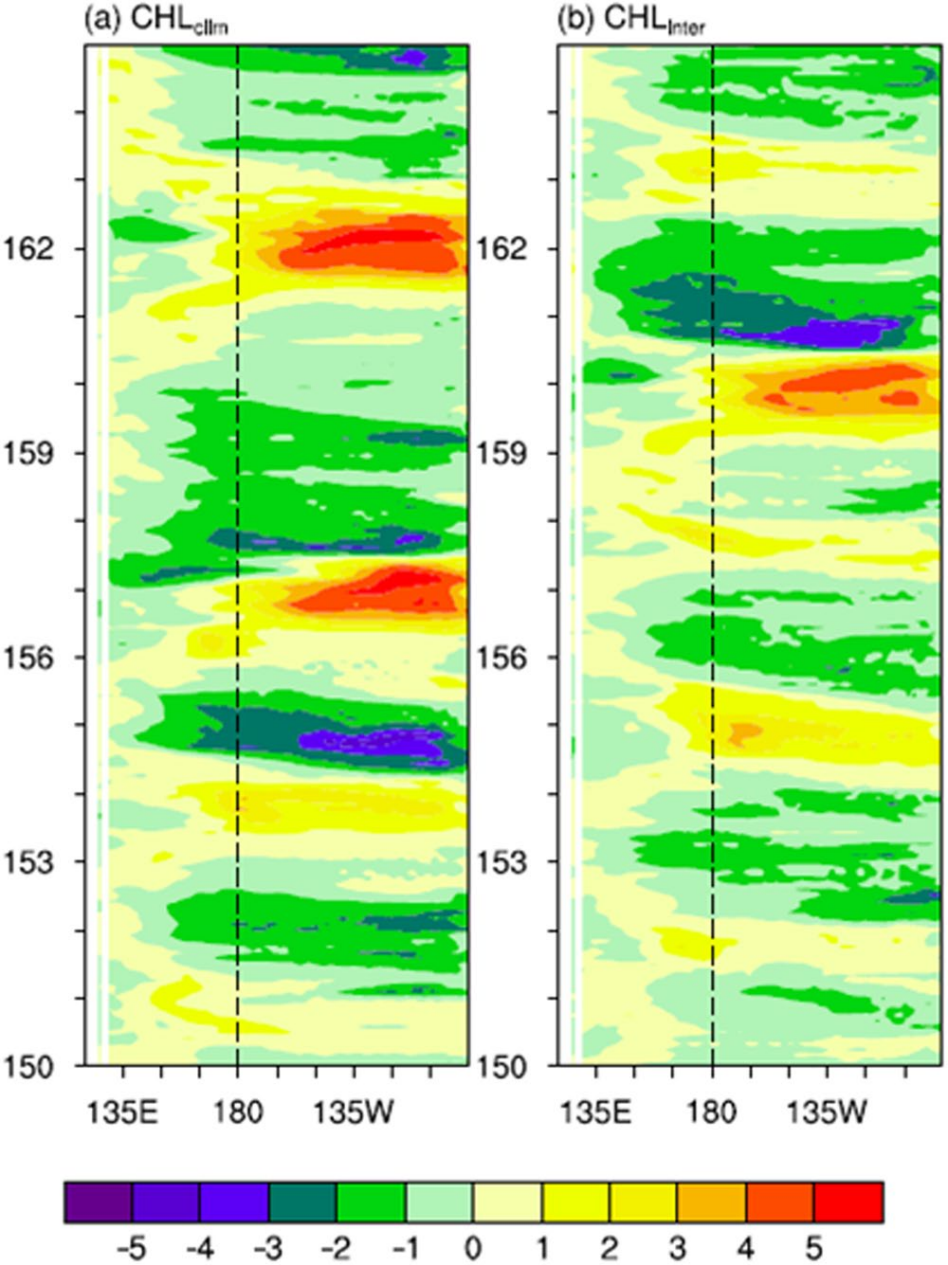
Longitude-time sections along the equator for interannual anomalies of SST simulated from the (**a**) CHL_clim_ and (**b**) CHL_inter_ runs. The anomalies are derived from subtracting monthly modeling SST by the monthly mean climatology (based on 151–200 modeling years). The contour interval is 1.0 °C.

**Figure 3:**
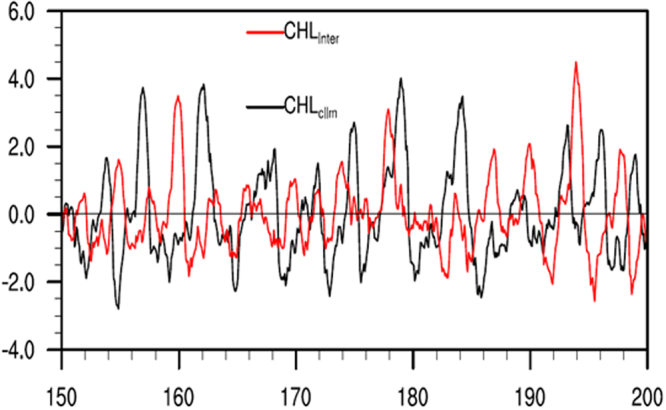
The Niño-3 index for the CHL_inter_ (red) and CHL_clim_ (black) runs.

**Figure 4:**
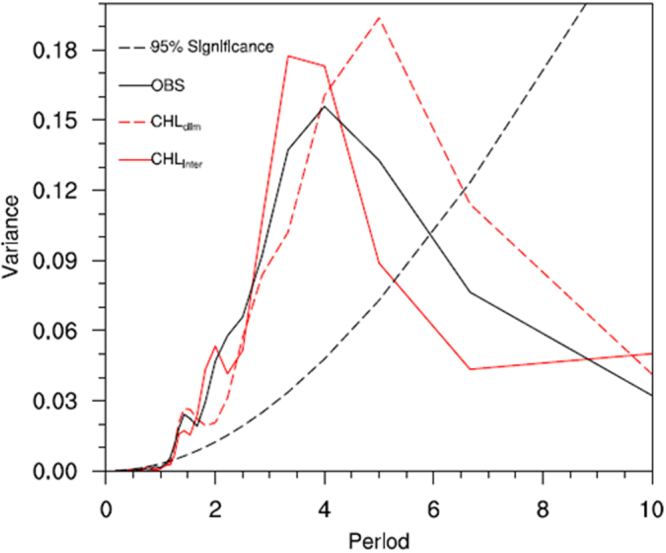
The power spectra for the Niño3 SST anomalies estimated for the observations (black), the CHL_inter_ run (red), and the CHL_clim_ (red dash) run. The dot-dashed line is the 95% significance level for these runs, assuming a white noise process.

It is evident that the model can well depict the mean ocean climatology and its variability in the tropical Pacific compared with observations. In this paper, our focus is on the effects on interannual variability. Figure 2 shows the interannual SST anomalies along the equator simulated from the non-feedback (CHL_clim_) and feedback (CHL_inter_) runs. The interannual variability exhibits irregular oscillations that are characterized by ENSO events. Compared with observations, the amplitude of the SST variability is significantly overestimated in the CHL_clim_ run. When the interannually varying CHL-induced feedback is included, the ENSO amplitude is significantly reduced, as indicated by the SST anomalies. To further demonstrate the effect of CHLAs on SSTAs, the area averaged SST index is shown in Fig. 3. The amplitude of ENSO in the CHL_inter_ run is substantially smaller than that in the CHL_clim_ run, especially during the first several decades of the 50-year integration period. The damping effect of CHLAs on ENSO occurs not only during El Niño events but also during La Niña events. The commonly used Niño3.4 SST series are used to quantify the dominant time scales of ENSO (Fig. 4). The interannual variability has a sharp peak at 4 years in the CHL_inter_ run compared with 5 years in the reference CHL_clim_ run, with a difference of approximately 12 months in the oscillation periods.

**Figure 5:**
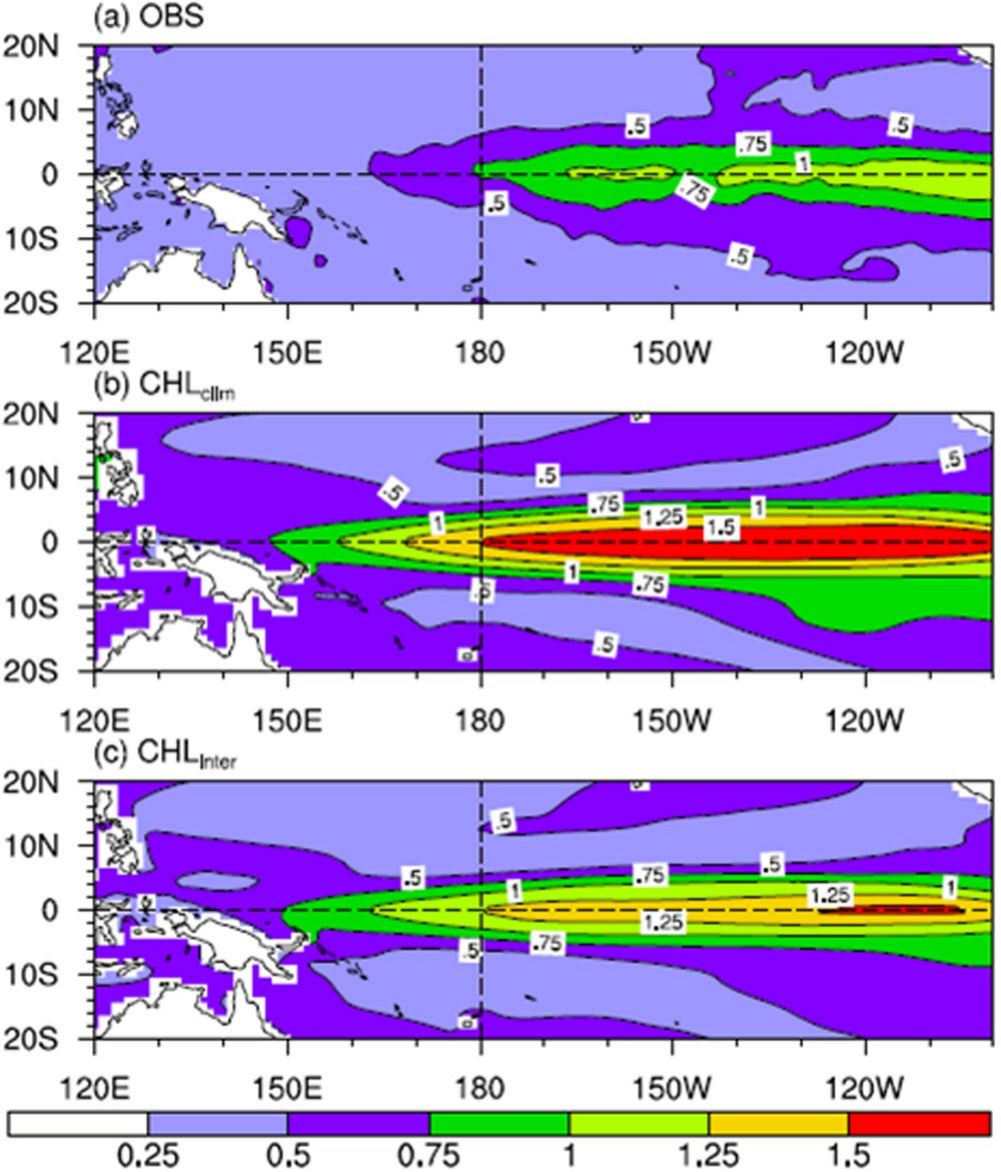
The standard deviation of SSTAs for (**a**) observations, (**b**) the CHL_clim_ run and (**c**) the CHL_inter_ run. The contour interval is 0.25 °C. The figure is created by the authors using the Grid Analysis and Display System (GrADS) which is available at http://www.iges.org/grads/grads.html.

Quantitatively, Fig. 5 shows the standard deviation of the SSTA for the observations and the CHL_clim_ and CHL_inter_ experiments. The standard deviation can be used as a metric for measuring ENSO amplitude. The strongest interannual variability of the observed SSTA appears in the eastern equatorial Pacific, which has a standard deviation exceeding 1.0 °C (Fig. 5a). In the CHL_clim_ run, the model well captures the spatial distribution of the SST variability; however, the amplitude of the variability is significantly overestimated, with the maximum standard deviation being over 1.5°C (Fig. 5b). This bias is a long-standing problem for the CESM, and it can be attributed to many factors^1,2^. When considering the interannually varying effect of CHLAs in the CHL_inter_ run, the SST amplitude bias can be effectively reduced, with the maximum standard deviation being reduced to 1.25°C (Fig. 5c). The area averaged standard deviation of SSTAs in the Niño3 and Niño4 regions is summarized in Table 2. In the Niño4 region, the standard deviation is 1.03 for the CHL_inter_ run and 1.22 for the CHL_clim_ run, whereas it is 0.71 for the corresponding observation. Thus, the inclusion of the interannual varying CHL effect leads to a damped ENSO amplitude. A similar reduction in the SST variability also occurs in the Niño3 region, where the standard deviation of SSTAs is 1.17 for the CHL_inter_ run and 1.46 for the CHL_clim_ run, and the standard deviation is 0.93 for the corresponding observation. These values represent decreases of approximately 34 and 43% for the Niño3 and Niño4 SST variabilities in the feedback run compared to the non-feedback run.

**Table 2:** The standard deviations of SST anomalies in the Niño4 and Niño3 regions from the CHL_clim_ and CHL_inter_ runs.

	Niño 4	Niño 3
OBS	0.71	0.93
CHL_clim_	1.22	1.46
CHL_inter_	1.03	1.17

Since the interannual variability in the tropical Pacific has a strong interaction with the mean states in the region, the effects on mean states and seasonal cycles are presented in the Supplementary section. It is evident that there is little change in the mean thermocline and seasonal cycle of SST in the equatorial Pacific due to the feedback of CHL and SST. For example, the seasonal cycle of SST in the eastern Pacific has been changed about 0.2 K when the CHL-induced feedback on SST is taken into account, with slight amplification of seasonal cycle of SST in the feedback (CHL_inter_) run (a warming in the spring and a cooling in the fall) compared with the non-feedback (CHL_clim_). Previous studies have suggested that an enhanced seasonal cycle tends to reduce interannual variability in the tropical Pacific^12^. It is unlikely that such a slight increase in the seasonal cycle in the feedback (CHL_inter_) run can be responsible for the reduction in the interannual variation of SST as seen in the two runs.

The processes responsible for the reduced ENSO amplitude as seen in the CHL_inter_ run are examined (the details are presented in the Supplementary section, including a calculation of the SST budget). CHL in the ocean affects the penetration of the incoming downward shortwave flux (SWDN) at the sea surface, and two heating terms are directly related with the bio-effects in the upper ocean. One is the SWDN component that is absorbed within the first layer (Q_abs_) and another is the SWDN component that penetrates into the subsurface layers through the bottom of the first layer (Q_pen_). It is found that the Q_abs_ and Q_pen_ fields are two terms that are directly modulated by interannual anomalies of CHL^19^. Therefore, the relationships among these related fields can be analyzed to understand the effects of the CHL-related feedback and the involved processes. As detailed in the Supplementary section, two pathways are possible for the variations in CHL that can affect SST in the equatorial Pacific, depending on the relative dominance of which term is modulated most pronouncedly, systematically, and coherently. On one hand, if a change in CHL induces a coherent and systematic change to Q_abs_, a direct heating effect on SST can be a dominant process that causes a corresponding change to SST; this is referred to as a direct effect. Then, it follows that the differences in CHL and Q_abs_ between the two runs should be well matched with each other. On the other hand, if a change in CHL causes a systematic and significant modulation of Q_pen_, differential heating can be then induced vertically between the first layer (Q_abs_) and subsurface layers (Q_pen_). These processes can further modify the stratification and vertical mixing in the upper ocean and thus affect SST; this is referred to as an indirect effect on SST. Therefore, the extent to which these two heating terms are affected by interannual CHL anomalies implies that different dominant processes are involved in the bio-effects. How Q_abs_ and/or Q_pen_ are affected by interannual CHL anomalies can be a good indicator of which influence pathways are taken and the underlying processes operating in association with the CHL-induced heating feedback in the NCAR CESM.

To identify the effect of the reduced CHL concentration on the total SWDN reaching the ocean surface, Q_abs_ and Q_pen_ are analyzed during the El Niño evolution in the feedback and non-feedback runs (the Supplementary section). Interannual CHL anomalies can have a direct effect on the total SWDN reaching the ocean surface and the vertical penetration in the upper ocean. It turns out that the total SWDN is not systematically and coherently affected by CHLAs. Similarly, the difference fields in Q_abs_ do not show coherent and systematic relationships with CHLAs. Indeed, the differences in Q_abs_ and SST between the two runs do not well match each other. As a result, the direct heating effects within the first layer induced by CHLAs (Q_abs_) cannot be a dominant process that causes the systematic differences in the SST simulations between these runs. In fact, the differences in Q_abs_ are negatively correlated with those in SST. This is consistent with the understanding that on interannual time scales associated with ENSO, heat flux and related direct solar radiation heating/cooling play a damping role on SST warming during El Niño evolution. As the SST difference between the two runs is spatially well defined, with consistent cooling (warming) in the eastern (western-central) equatorial Pacific (the Supplementary section), oceanic dynamical processes must play a dominant role in regulating SST variability in the tropical Pacific.

Indeed, it is found that although the difference in the total SWDN between the feedback and non-feedback runs is not systematic, the difference in Q_pen_ exhibits a well-defined and coherent pattern that is well matched with CHLAs (the Supplementary section). For example, a consistent increase in Q_pen_ is seen during El Niño events in the feedback run, which is accompanied with a low CHL concentration. The negative CHLAs increase penetration into the subsurface later throughout the bottom of the first layer (Q_pen_) and reduce the component that is absorbed within the first layer (Q_abs_). So, the increased Q_pen_ field warms the temperature of the subsurface layers. Such vertical redistribution of the absorbed SWDN (i.e., warming in subsurface layers but cooling in the first layer) acts to enhance the vertical mixing and further decrease the SST. As a result, the direct effect of SWDN absorbed in the first layer is unlikely to be a major contributor to the SST differences; instead, the indirect effect of the differential heating induced by Q_pen_ and Q_abs_ resulting from interannual anomalies of CHL can be a major contributor. As seen, relative to the non-feedback run, the warm SST anomalies in the feedback run are weakened due to the inclusion of CHLAs in the ocean, leading to a damping effect on the El Niño event.

In short, Q_pen_ is the term that is systematically affected by interannual CHL anomalies, and it can be responsible for the differences in the ENSO simulations seen in the feedback and non-feedback runs. Through the dynamic response induced by modulation of Q_pen_ in the upper ocean, the SSTs are modulated. Therefore, the SST difference between the two runs can be attributed to the effects of interannual CHL anomalies on Q_pen_. This is consistent with previous modeling studies that demonstrate that the CHL anomalies act to modulate ENSO mainly through modulation of solar radiation penetration throughout the bottom of the oceanic mixed layer^17,19^.

## Discussion

Recent studies indicate interactive coupling between ocean ecosystem and climate system on various space-time scales^24–26^. As a main focus in the tropical Pacific, ENSO has been extensively investigated; various observations-based analyses have demonstrated significant feedbacks of the ocean biology-induced heating on interannual variability^27–31^. Because interactions between ocean biology and physics are very complicated, numerical models provide a powerful tool to study the related feedback and coupling^32–34^. However, our current understanding of the influences of oceanic biology-induced heating feedback on ENSO and of the related physical processes remains elusive in ocean and climate models. In particular, the relationships between the existing ENSO biases in the NCAR CESM and how bio-feedback is represented are not well known. Previously, great efforts have focused on the physical aspects, but with less attention to the oceanic biological attributions. In this study, an SVD-based statistical model for interannual CHL anomalies is derived to identify its relationship with SST^23^, which is then incorporated into the NCAR CESM. We show that such an SVD-based statistical model can well reproduce the main characteristics of interannual CHL variability compared with observations. Numerical runs with and without the interannually varying CHLA effects are performed using the CESM and compared to each other. These results clearly demonstrate that climate biases in the current CESM can be greatly reduced by considering the bio-feedback. Specifically, the CHLA has obvious damping effects on ENSO by shortening the oscillation periods. To explain this, two more experiments are performed with a focus on one specific El Niño event. The difference fields between the two runs are analyzed to reveal dominant terms that are directly modulated by interannual CHL anomalies and thus the underlying processes responsible for the differences in ENSO simulations. The effects and related processes during the El Niño evolution are clearly illustrated in association with interannual CHL anomalies. Q_abs_ (the absorbed component of the incoming solar radiation in the first layer) and Q_pen_ (the component penetrating into the subsurface layers through the bottom of the first layer) are two terms that are directly affected by CHLAs. The differences in Q_abs_ between the two runs are not well matched with CHLAs; Q_abs_ is not systematically modulated by CHLAs, so it is not a major factor directly responsible for the SST difference. Instead, the Q_pen_ field is systematically modulated by CHLAs. The differences in CHL and Q_pen_ between the two runs are well matched with each other. As shown, the Q_pen_ field causes a dynamic response in the upper ocean, leading to modulations of SSTs. During an El Niño event, for instance, the negative CHLA allows solar radiation to penetrate more into the subsurface layers throughout the bottom of the first layer and to be absorbed less within the first layer. This directly leads to warming in the subsurface layers and cooling in the first layer. Furthermore, the temperature decrease in the first ocean model layer and temperature increase in the subsurface layers induce a vertical differential heating, which enhances vertical mixing and indirectly leads to SST decrease and so, the El Niño event is damped. As Q_pen_ is responsible for the damping effect on SST, the effects indirectly induced by CHLAs play a more dominant role in the damping effect on ENSO.

At present, obvious large biases exist in many of the state-of-the-art fully coupled models that explicitly include a biogeochemical component. For example, climatological CHL simulations still have large discrepancies in the tropical Pacific. In this study, an anomaly approach is taken in which the CHL climatological field is prescribed from observation, and its anomaly field is derived from SST anomalies. It is evident that the SVD-based statistical model for CHLAs is very efficient computationally and can well reproduce the main characteristics of the CHLAs compared to those of the observations. In this study, the feasibility and effectiveness of this statistical modeling approach are clearly justified by its success in improving ENSO simulations when this simple model is implemented in the NCAR CESM. It is expected that this simple model for interannual CHL variability can be used in other climate models to consider bio-feedback adequately, with climate simulations improved significantly.

This paper is intended to illustrate ENSO modulations that are induced by ocean biology-induced feedbacks within the tropical Pacific climate system. Numerical experiments are performed using the empirical model with the parameter representing feedback intensity that is tunable. Thus, the results presented in this paper are preliminary and need to be validated using more realistic and comprehensive models. For example, there is uncertainty/sensitivity in the represented amplitude of the bio-feedback to SST changes as indicated by the feedback strength factor *α*. In the current modeling study to reasonably represent the intensity of the OBH effects, *α* is doubled (Note that when taking *α* = 2 in the feedback simulation, the standard deviations of interannual H_p_ variability are well comparable to those from satellite-based estimates and thus, the OBH effect can be reasonably represented in the model simulation. Sensitivity experiments testing the effects of varying *α* on ENSO simulations are performed (Supplementary section). Indeed, within the statistical modeling context of H_p_, the choice of this parameter is rather arbitrary, and the modeling results are sensitive to the related parameters used. Additionally, a long term integration is also performed and the results are presented in Supplementary section.

## Methods

The physical model used in this study is the Community Earth System Model version 1.0.5 (CESM1.0.5) developed at the National Center for Atmospheric Research (NCAR); see the Supplementary section for details^21,22,35^. It is notable that the oceanic model in the NCAR CESM already includes a biogeochemical component that can be used to describe the biogeochemical processes and their interactions with the ocean physical system. Its performance, however, is still not accurate enough for practical uses. Additionally, the CESM with an interactive and explicit biogeochemical component included is extremely time consuming and impractical to run extensively. Instead, CHL is commonly prescribed to represent seasonally varying climatology derived from satellite data; we use this CHL setting as our reference for the CESM simulation (CHL_clim_), in which the oceanic biogeochemistry model is not activated, so there is no interannual bio-coupling between the oceanic physical model (POP2) and biogeochemical model. As such, the CHL_clim_ simulation does not include interannually varying CHL-induced climate feedback and the interactions between the physical and biogeochemical systems. As shown in this work, there are large biases in ENSO simulations when using this prescribed CHL_clim_ setting for the CESM.

Based on our previous work, we adopted a statistical modeling approach to represent the interannually varying CHL-induced climate feedback. This is based on the fact that on interannual time scales, ENSO is a major driver in the tropical Pacific, and interannual anomalies of CHL are represented as a response, which in turn can have feedback effects on ENSO. In particular, observed changes in ocean biology (e.g., CHL) closely follow physical changes (e.g., SST) during ENSO cycles. Based on the analysis conducted by Zhang *et al*.^23^, in this study, we adopted a singular value decomposition (SVD) method to capture the co-variations between the historical CHLA and SSTA during Sep. 1997-May 2016; a corresponding statistical model is then derived to relate the SSTA to the CHLA. Then, given an SSTA, the statistical model is used to calculate CHLAs with the first 5 SVD modes being retained, accounting for approximately 65% of the variance. This statistical model for CHLAs covers only the tropical Pacific (25°S-25°N, 124°E-76°W).

The derived statistical model for CHLAs is then incorporated into the CESM to represent CHL-induced feedback (Fig. 1). The total CHL field in the CESM can be described as

$$chl=ch{l}_{clim}+chla=ch{l}_{clim}+\alpha \cdot f(ssta)$$

where $\alpha$ is a scaling factor that is introduced to represent the amplitude of the derived CHLA, and *f* is a relationship between the interannual variations in SST and CHL that is determined using SVD analysis. In this way, the CHL in the CESM is composed of the seasonally varying climatological component ($ch{l}_{clim}$) and the interannually varying component ($chla$). $ch{l}_{clim}$ is prescribed from the historical satellite-based observations, and $chla$ is calculated at each ocean time step based on the instant SSTA through the SVD-based statistical model (Fig. 1). The SSTA is calculated at each ocean time step by subtracting the SST climatology field (SST_clim_) from the modeled SST (the SST_clim_ is derived from a long-term simulation with prescribed CHL climatology). As such, an anomaly coupling is adopted for the physical (SST) and biological (CHL) systems with the climatological CHL field being prescribed. When only the first five SVD modes are maintained in the statistical model, some variance is inevitably lost because the SVD modes contain only approximately 65% of the variance. In this study, $\alpha$ = 2.0 is taken so that the amplitude of the CHLA simulated from the statistical model can be comparable to the observations (Fig. S1 in the supplementary section). Note that such an SVD-based statistical model is only applied to the tropical Pacific.

Using this statistical method, interannually varying CHL-induced climate feedback can be considered in the NCAR CESM even though the ocean biogeochemical component is disabled. Thus, the net downward shortwave flux at the ocean surface (SWDN) from the atmospheric model is attenuated in the upper ocean due to the CHL field, which includes the interannual variation in the tropical Pacific. The vertically redistributed SWDN in the upper ocean acts to affect the SST. Interactively, the changed SST can also influence the atmosphere and the SWDN. Thus, the interactions between the CHLA and the physical system can be represented in the NCAR CESM (Fig. 1).

To assess the bio-effects, numerical experiments are conducted using the NCAR CESM (Table 1). First, the CESM with prescribed $ch{l}_{clim}$ is conducted for 150 years as a spin-up; then, two runs continue for an additional 50-year run (years 151–200). The CHL_clim_ run only includes the $ch{l}_{clim}$ field, and the CHL_inter_ run includes both the prescribed $ch{l}_{clim}$ and $chla$ fields derived from the SVD-based model (Table 1). The results from the CHL_clim_ and CHL_inter_ runs are analyzed and compared with each other. As the only differences in the two runs are interannual CHL variability included or not in the tropical Pacific, the effects of CHLAs on ENSO can be isolated in a clear way.

Additionally, two case studies are conducted to illustrate the detailed evolution of the related anomaly fields and the physical processes by focusing on a specific El Niño event that appears in the time integration during the model years 156–157 (Table 1). CHL_clim_–EN is a run in which CHL is used as its seasonally varying climatology; CHL_inter_–EN is a run in which interannually varying CHL effects are considered. These two runs are restarted from the same initial state at model year 153 and are time integrated for 2 years only. Detailed analyses are presented in the Supplementary section.

## Electronic supplementary material

Supplementary information
