# Supplementary material for: An improved ENSO simulation by representing chlorophyll-induced climate feedback in the NCAR Community Earth System Model
Source: Sci Rep. 2017 Dec 7;7:17123. doi: 10.1038/s41598-017-17390-2 (PMC5719437; doi:10.1038/s41598-017-17390-2)
Supplement: Supplementary file 1 — Supplementary information [file 41598_2017_17390_MOESM1_ESM.pdf]

Supplementary Material of

"An improved ENSO simulation by representing chlorophyll-induced climate feedback in the NCAR Community Earth System Model " by

Xianbiao Kang, Rong-Hua Zhang, Chuan Gao & Jieshun Zhu

S1. The NCAR CESM

The physical model used in this study is the Community Earth System Model version 1.0.5 (CESM1.0.5) developed at the National Center for Atmospheric Research (NCAR). It is a fully coupled Earth system model that includes atmospheric, oceanic, terrestrial, sea ice, land ice, and biogeochemical components, together with a coupler to coordinate the interplays among each component. The atmospheric model is version 5 of the Community Atmosphere Model (CAM5) that is run with the finite volume dynamical core (Neale et al. 2010). The oceanic model is the Parallel Ocean Program version 2 (POP2) (Smith et al. 2010). The atmospheric model has 30 vertical levels, with a zonal resolution of 1.9° and a meridional resolution of 2.5°. The oceanic model has a nominal horizontal resolution of 1° (gx1v6) and 60 vertical levels with a thickness of 10 m in the upper 150 m.

It is notable that the oceanic model in the NCAR CESM already includes a biogeochemical component that can be used to describe the biogeochemical processes and their interactions with the ocean physical system. Its performance, however, is still not accurate enough for practical uses. Additionally, the CESM with an interactive and explicit biogeochemical

component included is extremely time consuming and impractical to run extensively. Instead, CHL is commonly prescribed to represent seasonally varying climatology derived from satellite data. We use this CHL setting as our reference for the CESM simulation (CHL_{clim}), in which the oceanic biogeochemistry model is not activated, so there is no interannual bio-coupling between the oceanic physical model (POP2) and biogeochemical model. As such, the CHL_{clim} simulation does not include interannually varying CHL-induced climate feedback and the interactions between the physical and biogeochemical systems. As we will show below, there are large biases in ENSO simulations when using this prescribed CHL_{clim} setting for the CESM.

S2. Observed data used

Various observational data are used to derive statistical models for interannual CHL anomalies and to validate model simulations. Surface chlorophyll datasets are from 1998 to 2016 from the GlobColour project, which supplies continuous data sets for merged L3 Ocean Color products (including SeaWIFS, MODIS, MERIS and VIIRS sensors; see details at http://hermes.acri.fr/index.php) (Maritorena et al. 2010). Then, monthly CHL-1 data (CHL concentration (mg/m^3) for case 1 waters) are interpolated from (0.25°×0.25°) grids to the model grids. The SST fields are from Reynolds and Smith (1994).

S3. Interannual CHL variability simulated using the SVD-based statistical model

It is necessary to evaluate whether the SVD-based statistical model can reproduce characteristics of the CHLAs compared to those of the observations. Fig. s1 displays the observed and simulated interannual CHL anomalies. A high CHL concentration is observed

during the La Niña events, and a low CHL concentration is observed during the El Niño events. Satellite data reveal a close relationship between interannual anomalies of SST and CHL. Indeed, coherent relationships exist between variations in the oceanic biological and physical fields on interannual time scales. As a response to the warm SST anomalies in the CHL_{inter} run, the corresponding CHL decrease is consistent with what is observed during the El Niño events. For example, negative CHL anomalies are negatively correlated with warm SST anomalies.

When using the SVD-based statistical model, the standard deviation of the CHLA simulated in the CHL_{inter} run is comparable to that observed in the spatial distribution, where large values appear near the equatorial western Pacific warm pool and cold tongue (Fig. s2). However, values in the CHL_{inter} run are slightly greater than those observed in the western-central equatorial Pacific. This can be attributed to the fact that interannual SST anomalies simulated in the CESM are much too strong (Kang et al. 2014); the overestimated SSTAs cause the simulated CHLAs to also be large when using the statistical model CHLs.

Satellite-based observations indicate that CHLAs are negatively correlated with SSTAs in most regions of the tropical Pacific (figures not shown). Usually, warmer SST is accompanied by stable stratification in the upper ocean that is associated with weaker vertical mixing and less vertical nutrient supply from deeper ocean, causing less phytoplankton and thus weaker CHLA. Although it cannot explicitly capture the complicated oceanic biological processes related to the blooming of CHL, the SVD-based statistical model captures well the observed relationships between the SSTA and CHLA. However, the simulated CHLA in the CHL_{inter} run indicates higher SSTA-CHLA correlations compared to the observation (e.g., the maximum value of the correlation coefficients can exceed -0.8 in the model compared to -0.6 in the observation). This is understandable considering that the SVD-based model used in our study is empirically derived from the observed relationship between the CHLA and SSTA. Based on

the results of this analysis, we are confident that the SVD-based statistical model can well capture the main characteristics of the CHLA, so it is suitable for investigating the effects of CHLAs on ENSO in the NCAR CESM.

S4. The processes involved

The incoming shortwave radiation in the upper ocean (I) decreases exponentially with depth, as described by

$$I(z) = I_0 \times (A_1 e^{B_1 z} + A_2 e^{B_2 z})$$

where I_0 is the downward shortwave flux at the ocean surface (SWDN), and A_1 (A_2) and B_1 (B_2) are coefficients that depend on CHL (Ohlmann 2003). This formula can be used to calculate the SWDN component that is absorbed within the first layer (Q_{abs}) and another component that penetrates into the subsurface layers through the bottom of the first layer (Q_{pen}).

CHL in the ocean affects the penetration of solar radiation in the upper ocean; the Q_{abs} and Q_{pen} fields are two terms that are directly modulated by interannual anomalies of CHL. Therefore, the relationships among these related fields can be analyzed to understand the effects of the CHL-related feedback and the involved processes. Two pathways are possible for the variations in CHL that can affect SST in the equatorial Pacific, depending on the relative dominance of which term is modulated most pronouncedly, systematically, and coherently. On one hand, if a change in CHL induces a coherent and systematic change to Q_{abs}, a direct heating effect on SST can be a dominant process that causes a corresponding change to SST; this is referred to as a direct effect. Then, it follows that the differences in CHL and Q_{abs} between the two runs should be well matched with each other. On the other hand, if a change in CHL causes a systematic and significant modulation of Q_{pen}, differential heating can be then induced

vertically between the first layer (Q_{abs}) and subsurface layers (Q_{pen}). These processes can further modify the stratification and vertical mixing in the upper ocean and thus affect SST; this is referred to as an indirect effect on SST. Therefore, the extent to which these two heating terms are affected by interannual CHL anomalies implies different dominant processes that are involved in the bio-effects. How Q_{abs} and/or Q_{pen} are affected by interannual CHL anomalies can be a good indicator of which influence pathways are taken and the underlying processes operating in association with the CHL-induced heating feedback in the NCAR CESM.

It is notable that when the CHL_{clim} and CHL_{inter} runs are restarted from the same initial conditions, the phases of ENSO events can differ from each other in their subsequent evolutions. At the very beginning of the time integration, there are only small differences in these two runs. Such differences increase with time, and this can be attributed to many factors, including air-sea interactions that come into play in the tropical Pacific. Some time later, the differences in the phases of ENSO become increasingly larger in the two runs. When the phases of ENSO in the CHL_{inter} and CHL_{clim} runs are different, making use of the difference fields between these two runs can be meaningless for illustrating why ENSO differs when the CHLA effects are included in the CHL_{inter} run. Indeed, it is difficult to identify the underlying processes that may be responsible for the differences in the ENSO simulations in these two runs. In addition, the physical processes involved in the CHLA effect and air-sea interactions are intertwined together, making it impossible to clearly separate the effects of CHLAs on ENSO simulations from other effects in such a long-term integration.

One feasible way to isolate these processes is to focus on a specific El Niño event. For example, an El Niño event appears in years 153 and 154 of the model, and we can use the CESM to perform two more experiments to reveal the direct effects of interannual CHL variability on simulated fields and to illustrate the underlying processes responsible for the

differences in ENSO simulations (Table 1). One experiment is referred to as CHL_{clim}–EN, in which CHL is taken as its seasonally varying climatology, and the other is referred to as CHL_{inter}–EN, in which interannually varying CHL effects are considered; the interannual CHL anomalies are calculated using the statistical model from SST anomalies. These two runs are restarted from exactly the same initial state at model year 153 and are time integrated for two years, representing the developing phase of El Niño to its decaying phase (Fig. s3).

During such a short time integration, the phases of ENSO in the two runs are still quite similar (Fig. s2), and this can be considered as a situation in which air-sea interactions and other processes are not significantly contributing to differences in the simulations. Instead, the direct effects of the included CHL anomalies can be a major contributor to the difference between the two runs. Thus, it is reasonable to directly analyze the difference fields between the two runs and identify the explicit physical processes that can be responsible for the effects of CHLAs during the El Niño evolution.

Fig. s4 shows that for the CHL_{clim}-EN run, the El Niño event begins in April, peaks around December of the first year (year 153), and finally decays to a nearly neutral state around June of the following year (year 154). In the CHL_{inter}-EN run, the CHLA is first added to the CHL climatology in May of year 153, and the CESM is then integrated into July of the following year. Indeed, there is little difference in the Niño3 index between the two runs during the first several months, and this different gradually grows. The Niño-3 index for both runs exhibits a similar evolution. However, the influence of the CHLA on the El Niño event can be clearly discerned. For instance, the amplitude of the Niño-3 index in the CHL_{inter}-EN run is less than that in the CHL_{clim}-EN run. Additionally, it is evident that the El Niño event in the CHL_{inter}-EN run decays one month earlier than that in the CHL_{clim}-EN run, indicating the damping effects of CHLAs on the El Niño amplitude. As a result, the El Niño event in the CHL_{inter}-EN run exhibits

an early phase shift compared with the CHL_{clim}-EN run. As the El Niño event develops into the mature phase in December of the first year, the corresponding anomalies (e.g., SSTAs and CHLAs) become larger, and the bio-effects also increase.

We further investigate the effects on the SSTA evolution in the equatorial Pacific. In the CHL_{clim}-EN run, the El Niño-related warm SSTA appears in the equatorial Pacific early in year 153, peaks around the end of the first year (the maximum SSTA values exceeds 5.0°C), and finally returns to a neutral condition around June of the next year (Fig. s4a). When the bio-feedback is explicitly included in the CHL_{inter}-EN run, a similar El Niño evolution can be seen, including the timing of the onset and mature and decay phases (Fig. s4b). However, during the mature phase of this event, a notable difference between these two runs is that the maximum SSTA in the eastern equatorial Pacific in the CHL_{inter}-EN run is approximately 1.0°C less than that in the CHL_{clim}-EN run (Fig. s4c). Such a difference is more pronounced in the decaying phase of the event, and the SSTA in the eastern equatorial Pacific in the CHL_{inter}-EN run is approximately 3.0°C less than that in the CHL_{clim}-EN run.

Accompanying the evolution of the warm SSTA, the CHL in the CHL_{inter}-EN run decreases in the central-eastern equatorial Pacific. Such a decrease in CHL is more evident in the central equatorial Pacific (less than -0.25 mg/m^3) than that in the eastern equatorial Pacific (approximately -0.2 mg/m^3) (Fig. s5a). In response to the large warm SSTA (Fig. s4b), for example, the negative CHLAs are large during June of the first year and April of the next year but become nearly zero (Fig. s5a) when the SST returns to a near neutral state in the decaying months of this event (Fig. s4b). Evidently, the interannual anomalies of CHL induce a feedback on those in SST. Two factors can be related to the weakened warm SST anomalies in the two runs. One is associated with the decrease in the SWDN absorbed in the first model layer (Q_{abs}; Fig. s6a), and the other is associated with the increased SWDN penetrating to the bottom of the

first layer (Q_{pen}; Fig. s6b).

To identify the effect of the reduced CHL concentration on the total SWDN reaching the ocean surface, Q_{abs} and Q_{pen} are analyzed during the El Niño evolution. Interannual CHL anomalies have a direct effect on the total SWDN reaching to the ocean surface and the vertical penetration in the upper ocean. Fig. s5b shows the SWDN difference between the CHL_{inter}-EN and CHL_{clim}-EN runs. The total SWDN is not systematically and coherently affected by CHLAs. Similarly, the difference fields in Q_{abs} do not show coherent and systematic relationships with CHLAs. Indeed, the differences in Q_{abs} and SST between the two runs do not well match each other. As a result, the direct heating effects within the first layer induced by CHLAs (Q_{abs}) cannot be a dominant process that causes the systematic differences in the SST simulations between these runs. In fact, the differences in Q_{abs} are negatively correlated with those in SST (Fig. s6a). This is consistent with the understanding that on interannual time scales associated with ENSO, heat flux and related direct solar radiation heating/cooling play a damping role on SST warming during El Niño evolution. As the SST difference between the two runs is spatially well defined (Fig. s4b), with consistent cooling (warming) in the eastern (western-central) equatorial Pacific, dynamic oceanic processes must play a dominant role in regulating SST variability in the tropical Pacific.

Next, the direct effect on Q_{pen} is evaluated. Fig. s6b exhibits the Q_{pen} differences between the CHL_{inter}-EN and CHL_{clim}-EN runs. Although the difference in the total SWDN between the CHL_{inter}-EN and the CHL_{clim}-EN runs is not systematic (Fig. s5b), the difference in Q_{pen} exhibits a well-defined and coherent pattern that is well matched with CHLAs. A consistent increase in Q_{pen} is observed during years 153 and 154 in the CHL_{inter}-EN run (Fig. s6b). The positive CHLAs increase penetration into the subsurface later throughout the bottom of the first layer (Q_{pen}) and reduce the component that is absorbed within the first layer (Q_{abs}). More

specifically, during the El Niño event in year 153, for example, SWDN is absorbed less within the first ocean model layer in the CHL_{inter}-EN run relative to that in the CHL_{clim}-EN run (Fig. s6b). In contrast, the reduced CHL concentration allows the SWDN to penetrate more into the subsurface layers throughout the bottom of the first layer (systematic positive Q_{pen} differences).

So, the increased Q_{pen} field warms the temperature of the subsurface layers. Such vertical redistribution of the absorbed SWDN (i.e., warming in subsurface layers but cooling in the first layer) acts to enhance the vertical mixing and further decrease the SST. As a result, the direct effect of SWDN absorbed in the first layer is unlikely to be a major contributor to the SST differences; instead, the indirect effect of the differential heating induced by Q_{pen} and Q_{abs} resulting from interannual anomalies of CHL can be a major contributor. As seen, relative to the CHL_{clim}-EN run, the warm SST anomalies in the CHL_{inter}-EN run are weakened due to the inclusion of CHLAs in the ocean; inclusion of CHLAs in the ocean leads to a damping effect on the El Niño event.

It is notable that we have also conducted other case studies focusing on both El Niño and La Niña events. For example, a similar analysis is performed for a La Niña case. We find similar damping effects of positive interannual CHL anomalies on the La Niña event; similar physical processes are involved but with the opposite sense.

S5. The effects on the mean ocean climatology

The model can well depict the observed mean SST climatology (Figs. s7a-b) and subsurface temperature (Figs. s8a-b) in the tropical Pacific. Also, the mean ocean states simulated are similar to each other in the different model experiments with the related OBH feedbacks being included or not (Figs. s7b-c; Figs. s8b-c) because their effects are of opposite signs between cold and warm phases of ENSO (Fig. 2 and Figs. s5-s6). Without the OBH feedbacks, the model can well reproduce the seasonal cycles of SST in the tropical Pacific which is calculated as the deviation of monthly climatology relative to its mean climate (Figs. s9a-b). The OBH feedbacks have little impact on seasonal cycles, which can be seen from the differences between CHL_{clim} and CHL_{inter} runs (Fig. s9c) that are small. Based on these analyses, it is evident that the mean climatology and seasonal cycles have not been influenced substantially by the OBH feedbacks. Thus, the reduced interannual variability associated with ENSO due to the included OBH feedbacks is unlikely resulted from changes in mean climatology and seasonal cycles, and instead is mainly attributed to physical processes discussed in the main text.

S6. The sensitivity to the intensity of ocean biology-induced feedback

This paper is intended to illustrate ENSO modulations that are induced by ocean biology-induced feedbacks within the tropical Pacific climate system. Numerical experiments are performed using the empirical model for interannual Chl anomalies with the parameter (α) representing feedback intensity that is tunable. As such, there is uncertainty/sensitivity in the represented amplitude of the bio-feedback to SST changes as indicated by the feedback strength factor α. For instance, to reasonably represent the intensity of the OBH effects, α is doubled in the current modeling study (Note that when taking α=2 in the feedback simulation, the standard deviations of interannual CHLA variability are well comparable to those from

satellite-based estimates [23] and thus, the OBH effect can be reasonably represented in the model simulation). Indeed, within the statistical modeling context of CHLA, the choice of this parameter is rather arbitrary, and the modeling results are sensitive to the related parameters used. To investigate the sensitivity of modeling results to the scaling factor (α), some experiments with different values of α (α=1.0, α=2.5) are further conducted and analyzed together with the CHL_{clim} (α=0.0) run and CHL_{inter} (α=2.0) run. Fig. s10 shows the the bias of the standard deviation for SSTAs with different scaling factors. With the increasing of feedback intensity (α increases from 0.0 to 2.5), the differences in the standard deviation decrease coherently. Although the differences can be decreased effectively by increasing the feedback intensity, it is not reasonable to match modeling results with observation by unlimited increasing of the values of scaling factor (α), because the CHLA retrieved is comparable to the observation only with α=2.0, and the modeling runs with too large values of α would produce an unrealistic CHLA distribution. With the damping effects of CHLA on ENSO, the period of ENSO acts to be shortened as demonstrated in Fig. 4. Such shortening effects are seen to be more significant if increasing the feedback intensity (Fig. s11).

S7. Long-term model runs

Some extended integrations (150 years) were performed using the model with the related OBH feedbacks being included or not. The longer simulations allow the model to have enough ENSO cycles. As shown in Fig. s12, in most time periods of the long-term integrations, the OBH feedbacks act to damp the interannual variability associated with ENSO. This can also been seen in Fig. s13, in which the differences in the standard deviation for SSTAs in CHL_{inter} runs is commonly smaller than that in CHL_{clim} runs. Also, the OBH feedback acts to shorten the period of ENSO, with the peak period in the CHL_{inter} runs about 12 months shorter than that in the CHL_{clim} run (Fig. s14). So, results from the

long-term simulations are in accordance with simulations analyzed from the shorter time integration (50 years) in the main text, clearly demonstrating that the effects of OBH feedbacks on ENSO properties do not change significantly with the length of integration time. Additionally, no significant difference (less than 0.05°C) can be found in the mean SST climatology between the CHL_{inter} and CHL_{clim} runs (Fig. s15). This can be attributed to the fact that the effects of CHLA on ENSO are of opposite signs between the two different phases (decreasing the SST in an El Niño event but increasing SST in a La Niña event), and their net compensating effects on SST climatology can thus be small.

S8. Heat budget analysis

For the first model layer, the heat budget can be written as

$$\frac{\partial T}{\partial t} = -\mathrm{V} \cdot \nabla T + \frac{HFLX}{\rho C_p h} + \frac{Q_{abs}}{\rho C_p h} + R,$$

where $\frac{\partial T}{\partial t}$ is the temperature tendency of the first model layer; V denotes three-dimensional ocean current; $\nabla = (\partial/x, \partial/y, \partial/z)$ represents a three-dimensional gradient operator; ρ is the density of sea water; C_p denotes the specific heat of sea water; h is the depth of the first model layer (10 m); $HFLX$ denotes the surface heat flux which includes the net longwave radiation flux, the sensible and latent heat flux; Q_{abs} indicates the total SWDN absorbed within the first model layer; and R represents the vertical mixing. Using the model outputs in both the CHL_{clim}-EN and CHL_{inter}-EN runs, each term contributing to the temperature tendency of the first model layer can be calculated and compared with each other. It is found that no significant differences exist for the advective term $(-\mathrm{V} \cdot \nabla T)$ and surface heat flux forcing $(\frac{HFLX}{\rho C_p h})$ between the CHL_{clim}-EN and CHL_{inter}-EN runs, and the differences of heat budget between both experiments are mainly attributed to the effects associated with the absorbed SWDN heating

($\frac{Q_{abs}}{\rho C_p h}$) and vertical mixing ($R$). Compared to that in the CHL_{clim}-EN run, the direct heating effect of the absorbed SWDN in the CHL_{inter}-EN experiment is relatively smaller (Fig. s16a) and the vertical mixing cooling effect is relatively stronger (Fig. s16b). So, the budget analyses support previous arguments about the CHL-related feedback. When CHL-related feedback is included, for instance, the reduced CHL during El Nino (Fig. s5) acts to allow less SWDN absorbed within the first model layer (Fig. s6a), but more SWDN penetrated to deeper layers (Fig. s6b). The reduced absorbed SWDN in the first model layer tends to directly decrease SST and the vertical thermal differential heating (cooling in the first model layer and warming in the subsurface layers) acts to strengthen the vertical mixing, which further decreases the SST.

Figure captions for the Supplementary section

Fig. s1 Longitude-time sections along the equator for interannual anomalies of CHL (a) observed and (b) simulated using the statistical model. The contour interval is 0.05 mg/m^3.

Fig. s2 The standard deviation of CHLAs for (a) observations and (b) the CHL_{inter} run. The contour interval is 0.02 mg/m^3. The figure is created by the authors using the Grid Analysis and Display System (GrADS) which is available at http://www.iges.org/grads/grads.html

Fig. s3 The Niño3 index for the CHL_{inter}-EN (red) and CHL_{clim}-EN (black) runs.

Fig. s4 Longitude-time sections along the equator for SSTAs in (a) the CHL_{clim}-EN run, (b) the CHL_{inter}-EN run and (c) their differences (CHL_{inter}-EN – CHL_{clim}-EN). The contour interval is 0.5°C.

Fig. s5 Longitude-time sections along the equator for (a) CHLAs in the CHL_{inter}-EN run and (b) the difference in the SWDN between the CHL_{inter}-EN and CHL_{clim}-EN runs. The contour interval is 0.1 mg/m^3 in (a) and 15 W in (b).

Fig. s6 Longitude-time sections along the equator for the differences between the CHL_{inter}-EN and CHL_{clim}-EN in (a) the SWDN absorbed within the first model layer (Q_{abs}) and (b) the SWDN that penetrated throughout the bottom of the first model layer (Q_{pen}). The contour interval is 15 W.

Fig. s7 Annual-mean SSTs (°C) over the tropical Pacific: (a) Observations; (b) CHL_{clim}; (c) the difference between CHL_{inter} and CHL_{clim}. Observations correspond to the 1981–2010 average from the OI.v2 analysis (https://www.esrl.noaa.gov/psd/data/gridded/data.noaa.oisst.v2.html), and the CHL_{clim} and CHL_{inter} correspond to the model years 151-200. The contour interval in (a) and (b) is 1.0°C, and in (c) is 0.1°C. The figure is created by the authors using the Grid Analysis and Display System (GrADS) which is available at http://www.iges.org/grads/grads.html.

Fig. s8 Annual-mean ocean temperature (°C) in the thermocline along the equatorial Pacific: (a) OBS; (b) CHL_{inter}; (c) the difference between CHL_{inter} and CHL_{clim}. Observations show the results averaged during 1981–2010 from the NCEP Global Ocean Data Assimilation System (http://www.cpc.ncep.noaa.gov/products/GODAS/index.shtml), and model results from the CHL_{clim} and CHL_{inter} simulations are averaged during the model years 151-200. The contour interval is 2.0°C in (a) and (b), and is 0.2°C in (c).

Fig. s9 Seasonal cycle of SSTs along the equatorial Pacific region (averaged over 5°S-5°N): (a) Observations; (b) CHL_{clim}; (c) the difference between CHL_{inter} and CHL_{clim}. The mean climatological field is removed. The contour interval is 0.5°C in (a) and (b), and is 0.1°C in (c).

Fig. s10 The bias of the standard deviation for SSTAs with different scaling factors: (a) α=0.0; (b) α=1.0; (c) α=2.0; (d) α=2.5. The contour interval is 0.2°C. The figure is created by the authors using the Grid Analysis and Display System (GrADS) which is available at http://www.iges.org/grads/grads.html.

Fig. s11 The power spectra for the Niño3 SST anomalies estimated from model runs with different scaling factors: α=0.0 (red), α=1.0 (purple), α=2.0 (green), α=2.5 (blue). The dot-dashed line is the 95% significance level for these runs, assuming a white noise process.

Fig. s12 The Niño3 index for the CHL_{inter} (red) and CHL_{clim} (black) from long-term runs (150-300 years).

Fig. s13 The differences in the standard deviation of SSTAs from long-term runs (150-300 years): (a) CHL_{clim}; (b) CHL_{inter}. The contour interval is 0.25 °C。 The figure is created by the authors using the Grid Analysis and Display System (GrADS) which is available at http://www.iges.org/grads/grads.html.

Fig. s14 The power spectra of the Niño3 SST anomalies from long-term runs (150-300 years):

CHL_{clim} (blue) run, the CHL_{inter} run (green). The dot-dashed line is the 95% significance level for these runs, assuming a white noise process.

Fig. s15 The difference in the annual-mean SSTs over the tropical Pacific between the CHL_{clim} run and the long-term run (150-300 years). The contour interval is 0.05 °C. The figure is created by the authors using the Grid Analysis and Display System (GrADS) which is available at http://www.iges.org/grads/grads.html.

Fig. s16 Longitude-time sections along the equator for the differences between the CHL_{inter}-EN and CHL_{clim}-EN in (a) the contributions of the absorbed SWDN ($\frac{Q_{abs}}{\rho C_p h}$) to the temperature tendency of the first model layer and (b) the effect of vertical mixing (R) on the temperature tendency of the first model layer. The contour interval is 1.0×10^{-6} °C/s.

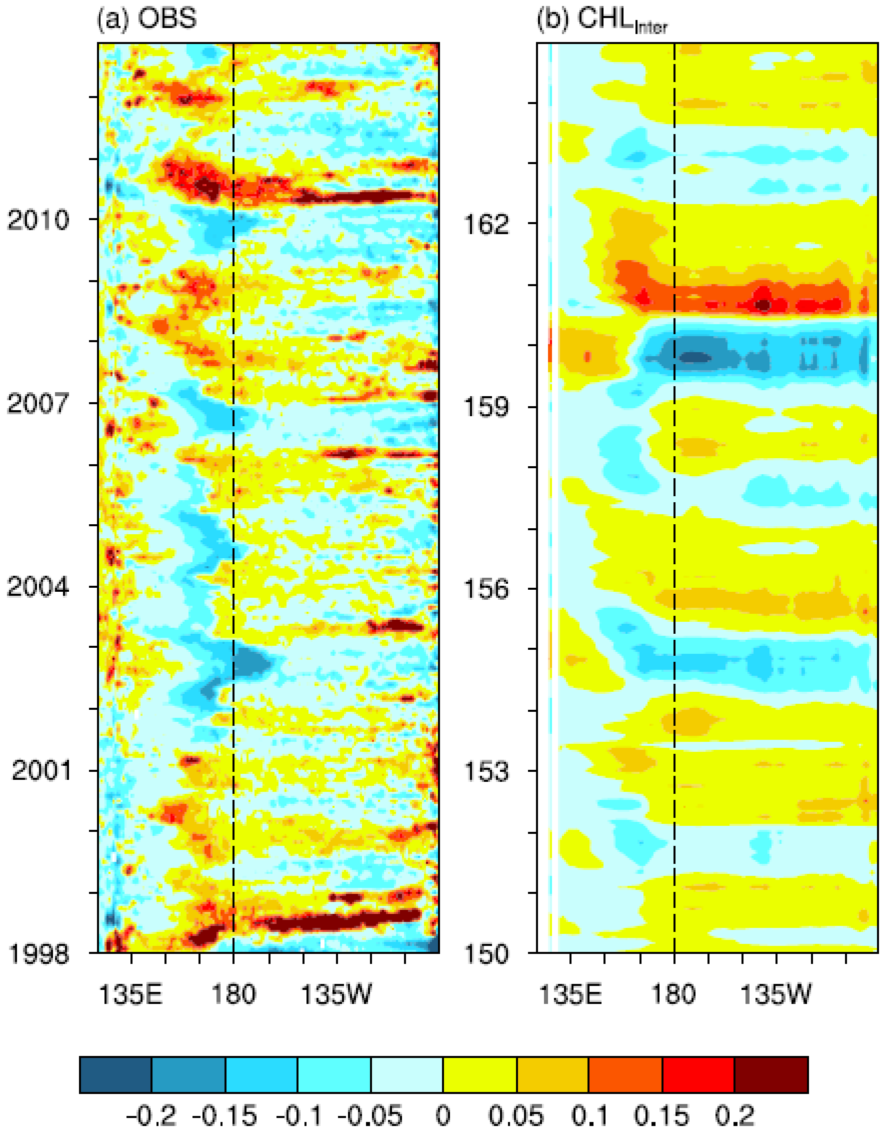

Fig. s1 Longitude-time sections along the equator for interannual anomalies of CHL (a) observed and (b) simulated using the statistical model. The contour interval is 0.05 mg/m^3.

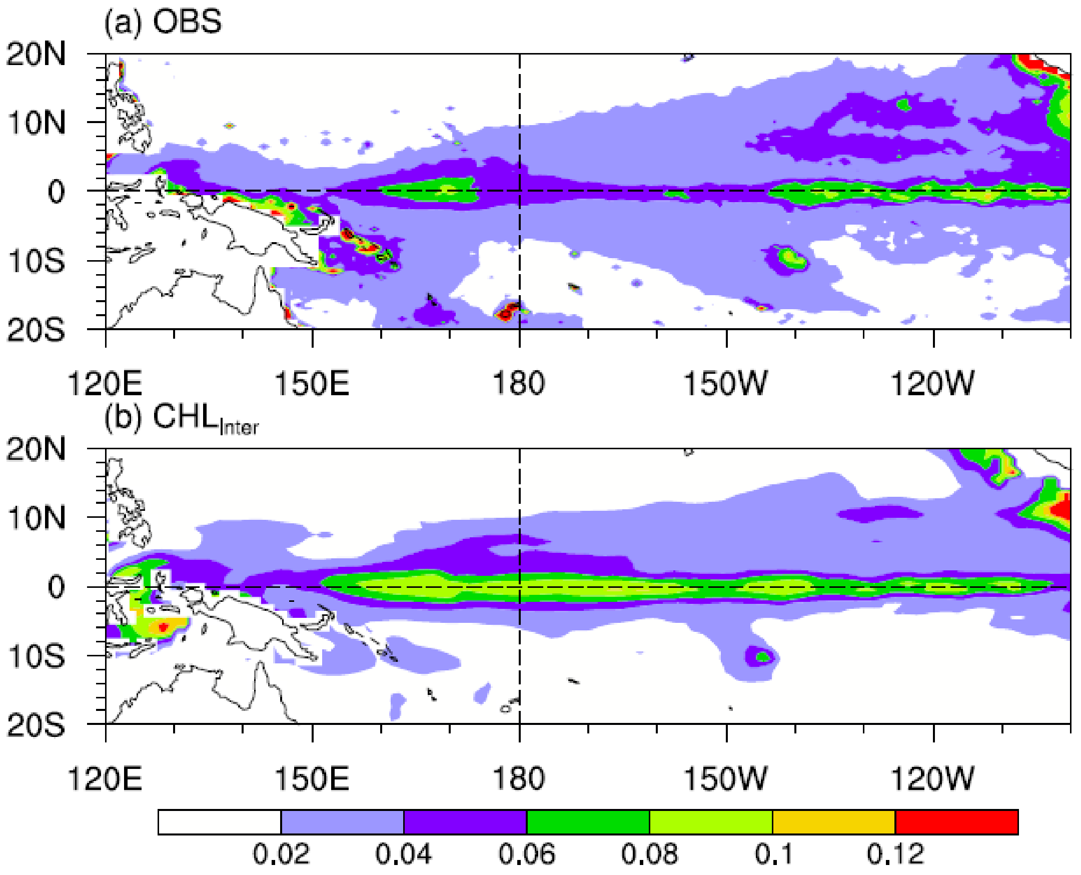

Fig. s2 The standard deviation of CHLAs for (a) observations and (b) the CHL_{inter} run. The contour interval is 0.02 mg/m^3. The figure is created by the authors using the Grid Analysis and Display System (GrADS) which is available at http://www.iges.org/grads/grads.html

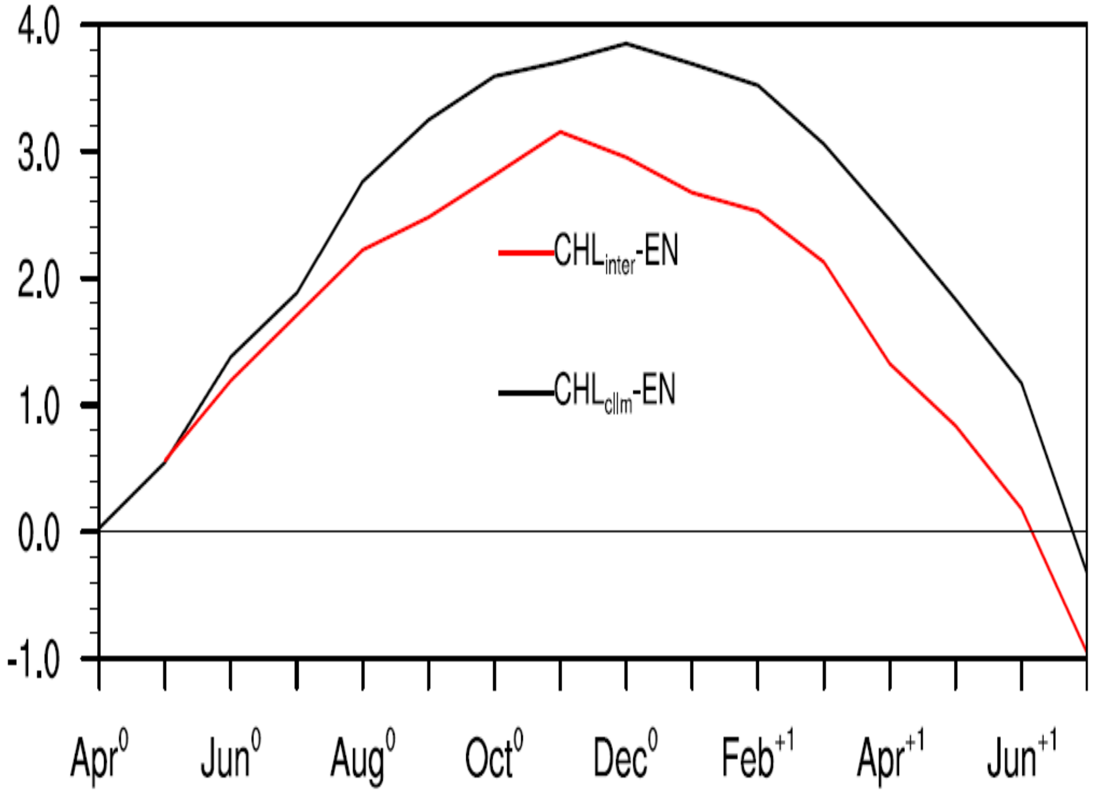

Fig. s3 The Niño3 index for the CHL_{inter}-EN (red) and CHL_{clim}-EN (black) runs.

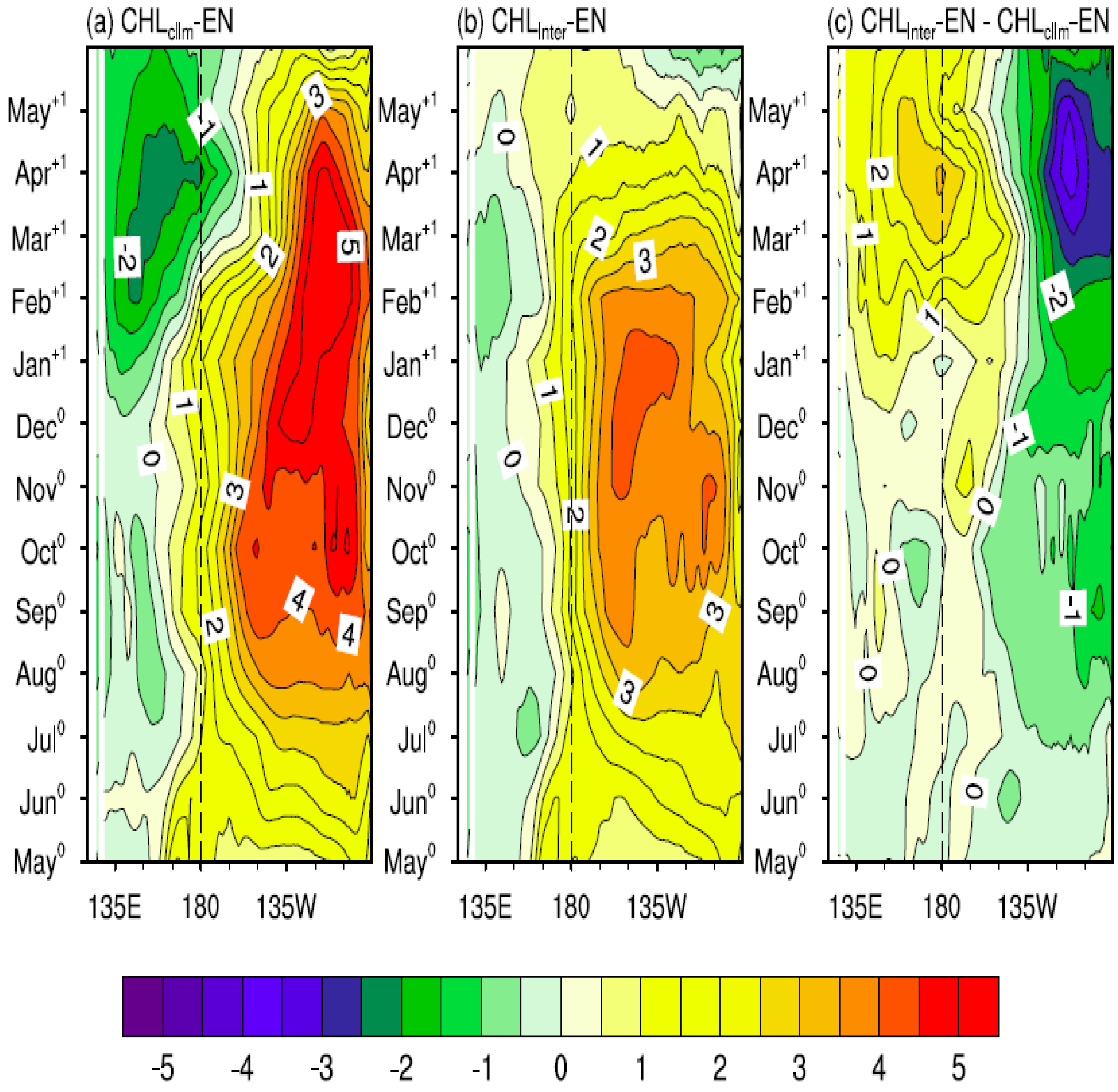

Fig. s4 Longitude-time sections along the equator for SSTAs in (a) the CHL_{clim}-EN run, (b) the CHL_{inter}-EN run and (c) their differences (CHL_{inter}-EN – CHL_{clim}-EN). The contour interval is 0.5°C.

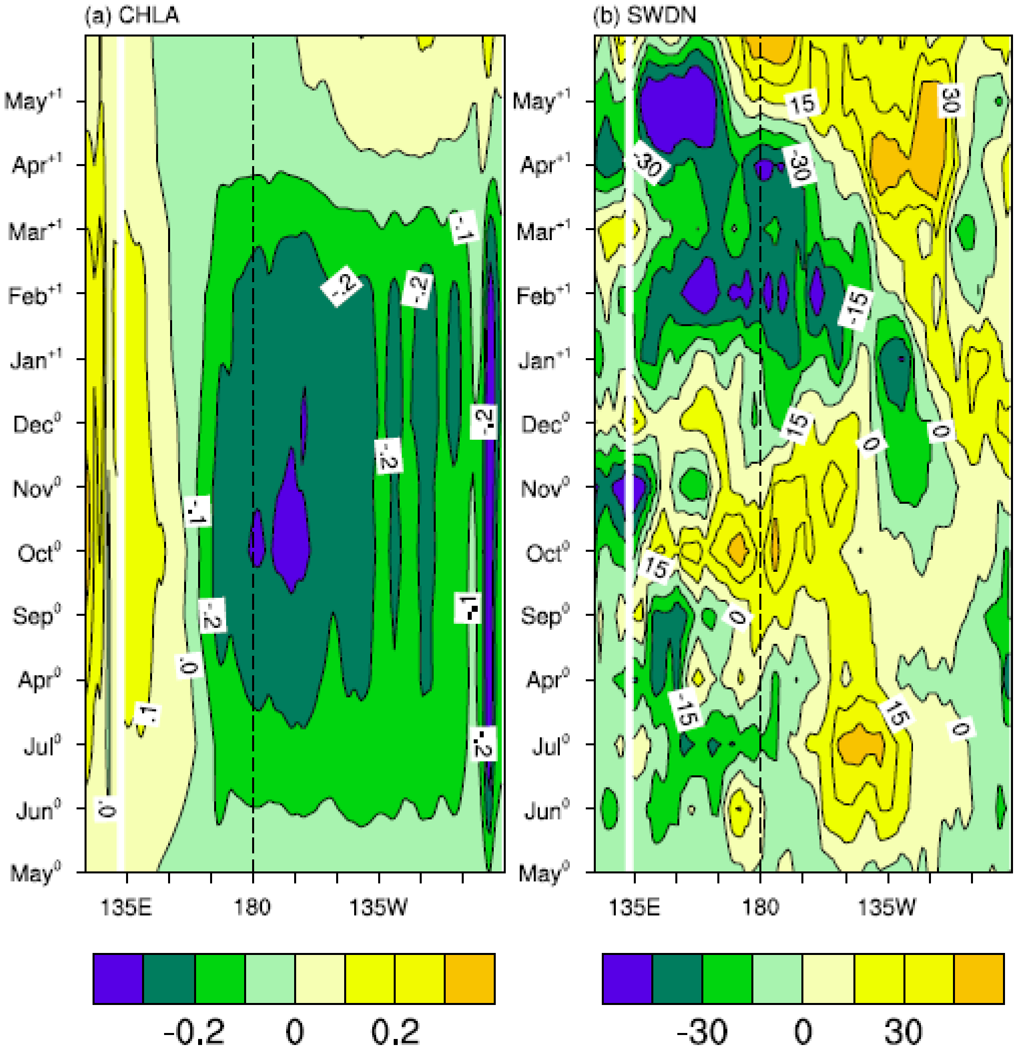

Fig. s5 Longitude-time sections along the equator for (a) CHLAs in the CHL$_{inter}$-EN run and (b) the difference in the SWDN between the CHL$_{inter}$-EN and CHL$_{clim}$-EN runs. The contour interval is 0.1 mg/m^3 in (a) and 15 W in (b).

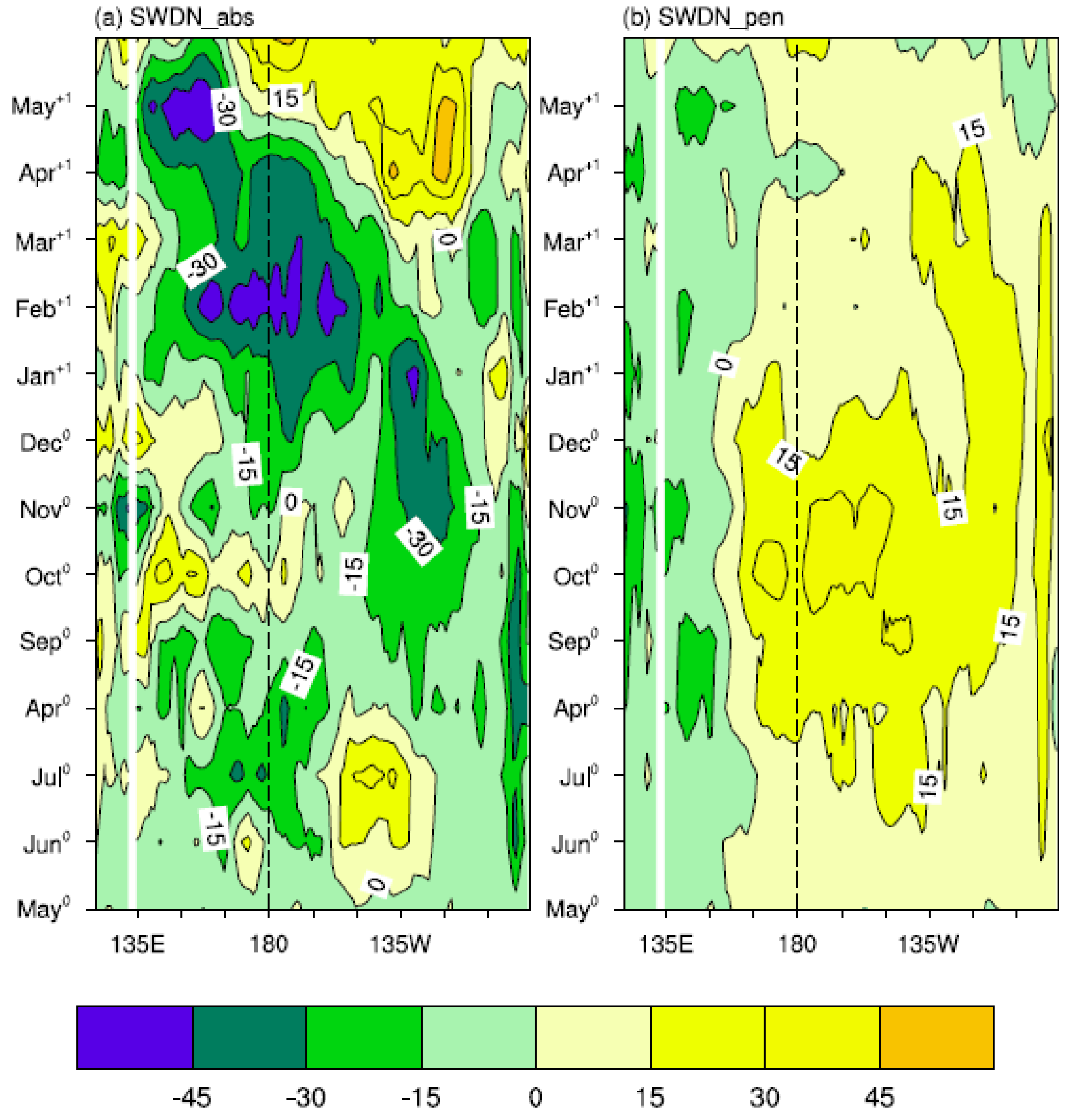

Fig. s6 Longitude-time sections along the equator for the differences between the CHL_{inter}-EN and CHL_{clim}-EN in (a) the SWDN absorbed within the first model layer (Q_{abs}) and (b) the SWDN that penetrated throughout the bottom of the first model layer (Q_{pen}). The contour interval is 15 W.

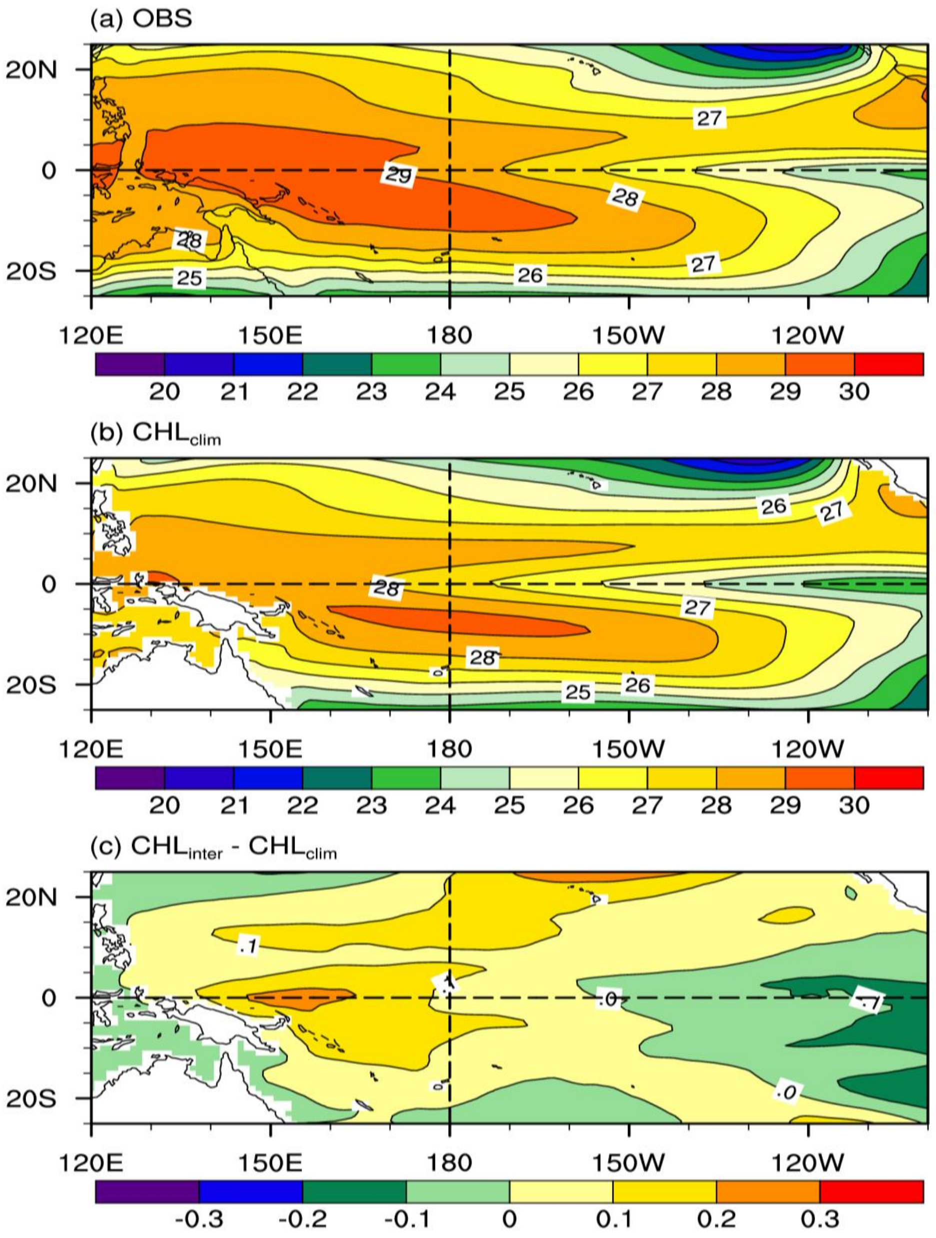

Fig. s7 Annual-mean SSTs (°C) over the tropical Pacific: (a) Observations; (b) CHL_{clim}; (c) the difference between CHL_{inter} and CHL_{clim}. Observations correspond to the 1981–2010 average from the OI.v2 analysis (https://www.esrl.noaa.gov/psd/data/gridded/data.noaa.oisst.v2.html), and the CHL_{clim} and CHL_{inter} correspond to the model years 151-200. The contour interval in (a) and (b) is 1.0°C, and in (c) is 0.1°C. The figure is created by the authors using the Grid Analysis and Display System (GrADS) which is available at http://www.iges.org/grads/grads.html.

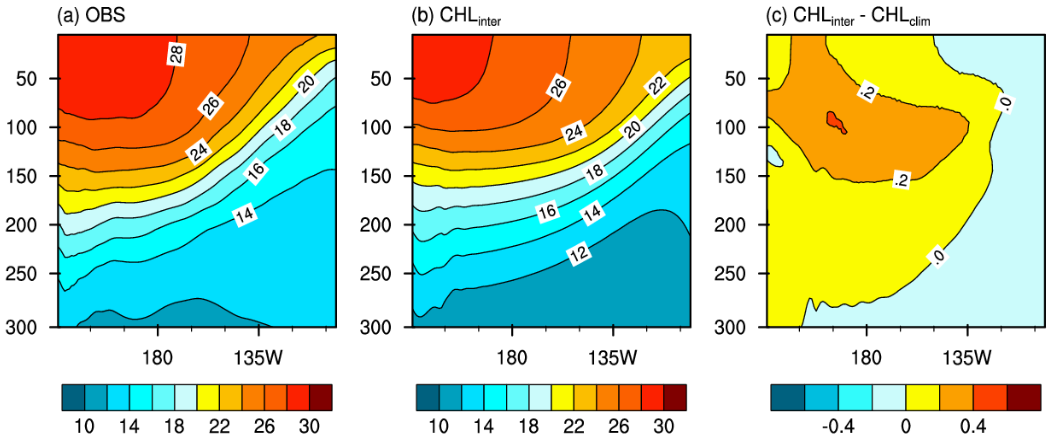

Fig. s8 Annual-mean ocean temperature (°C) in the thermocline along the equatorial Pacific: (a) OBS; (b) CHL_{inter}; (c) the difference between CHL_{inter} and CHL_{clim}. Observations show the results averaged during 1981–2010 from the NCEP Global Ocean Data Assimilation System (http://www.cpc.ncep.noaa.gov/products/GODAS/index.shtml), and model results from the CHL_{clim} and CHL_{inter} simulations are averaged during the model years 151-200. The contour interval is 2.0°C in (a) and (b), and is 0.2°C in (c).

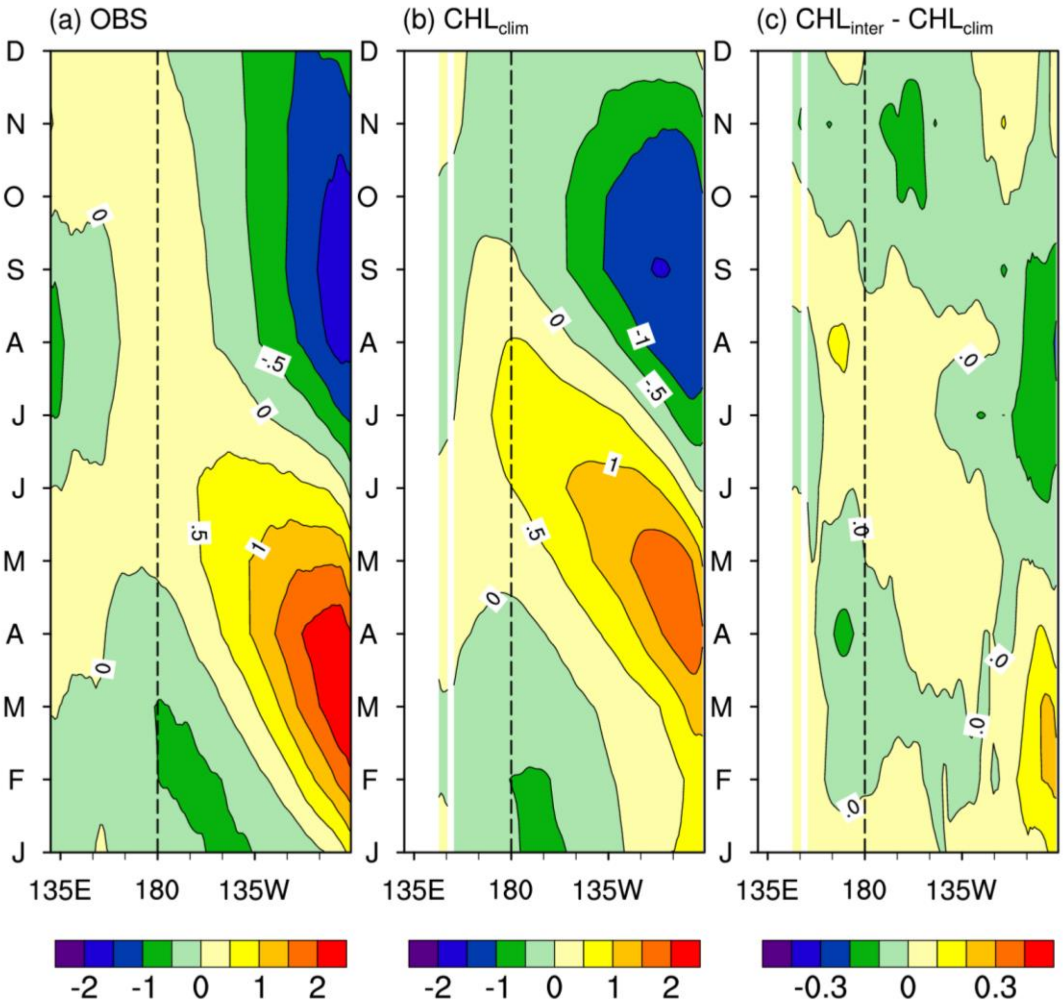

Fig. s9 Seasonal cycle of SSTs along the equatorial Pacific region (averaged over 5°S-5°N): (a) Observations; (b) CHL_{clim}; (c) the difference between CHL_{inter} and CHL_{clim}. The mean climatological field is removed. The contour interval is 0.5°C in (a) and (b), and is 0.1°C in (c).

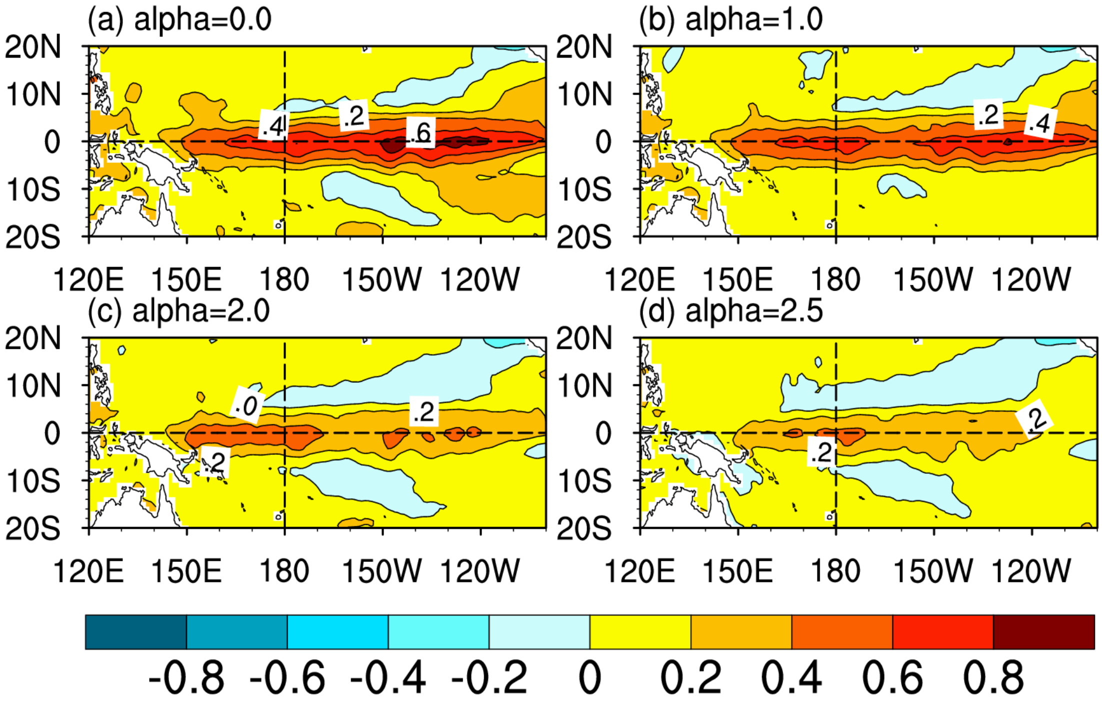

Fig. s10 The bias of the standard deviation for SSTAs with different scaling factors: (a) α=0.0; (b) α=1.0; (c) α=2.0; (d) α=2.5. The contour interval is 0.2°C. The figure is created by the authors using the Grid Analysis and Display System (GrADS) which is available at http://www.iges.org/grads/grads.html.

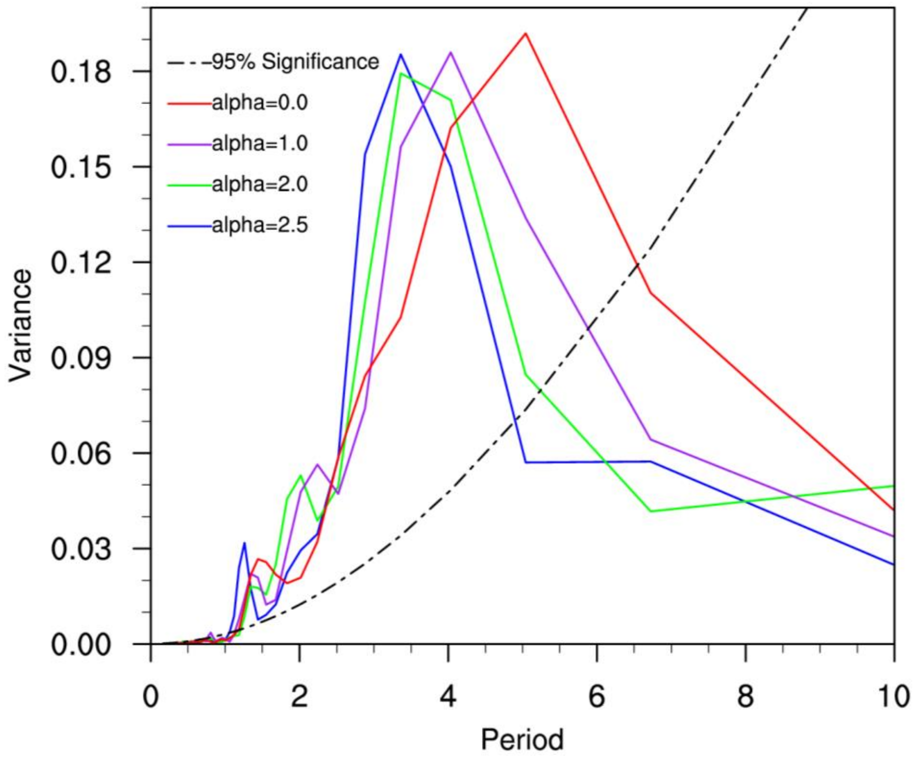

Fig. s11 The power spectra for the Niño3 SST anomalies estimated from model runs with different scaling factors: α=0.0 (red), α=1.0 (purple), α=2.0 (green), α=2.5 (blue). The dot-dashed line is the 95% significance level for these runs, assuming a white noise process.

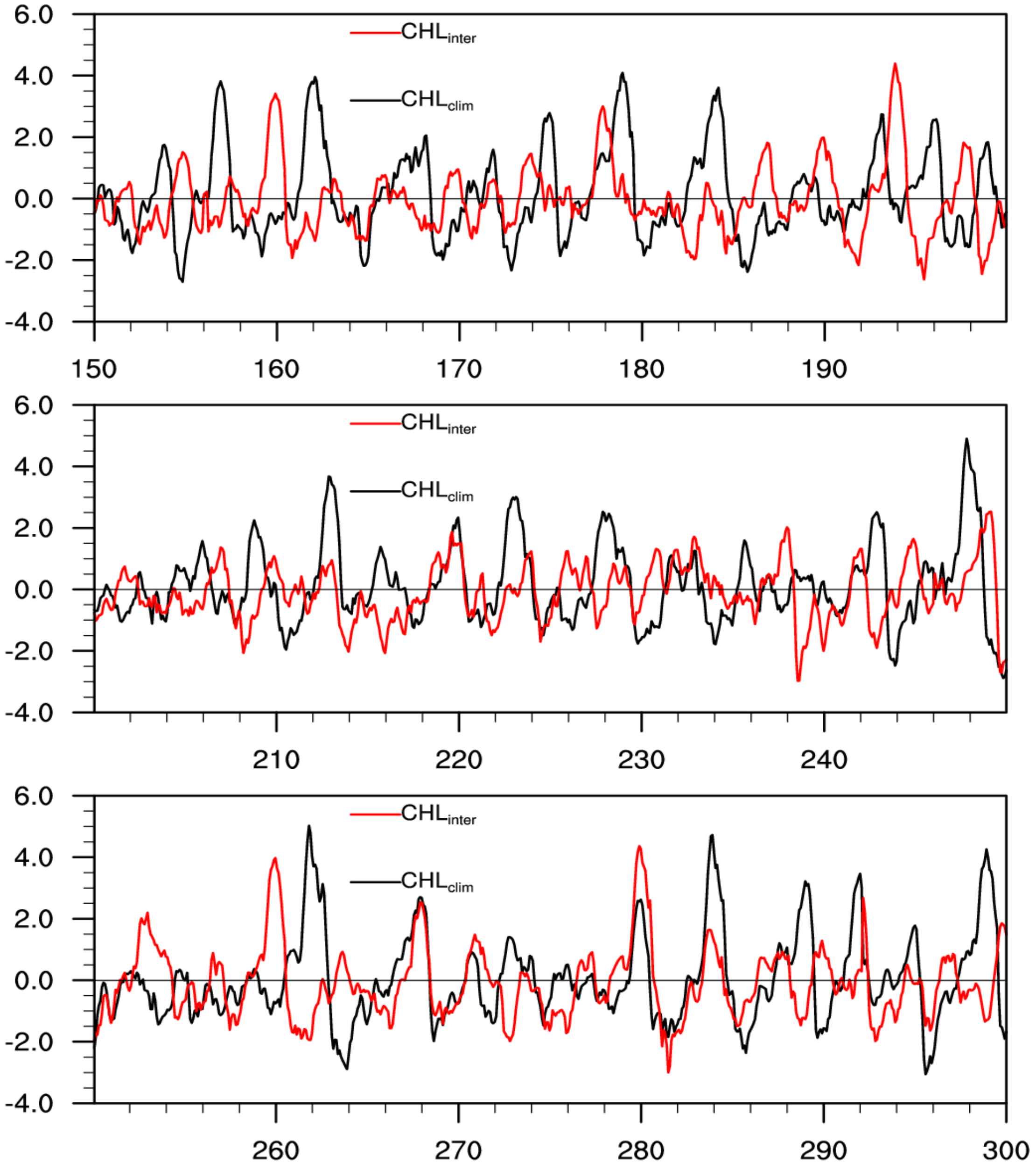

Fig. s12 The Niño3 index for the CHL$_{inter}$ (red) and CHL$_{clim}$ (black) from long-term runs (150-300 years).

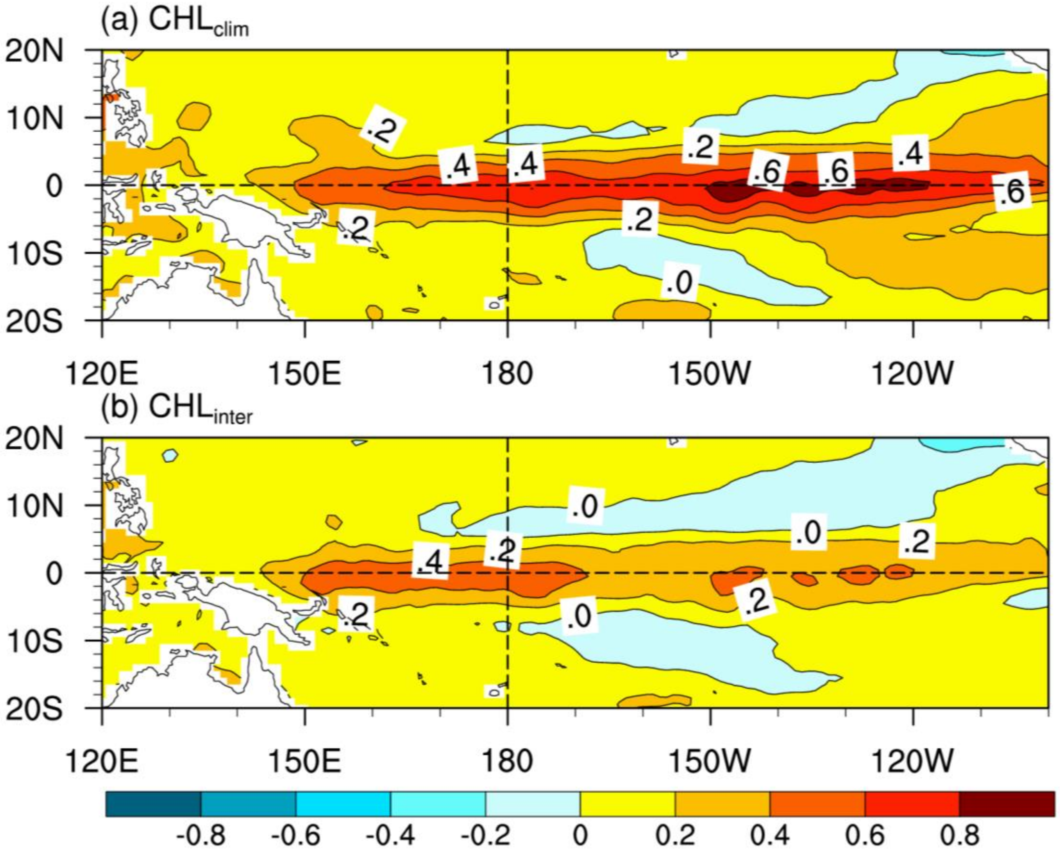

Fig. s13 The differences in the standard deviation of SSTAs from long-term runs (150-300 years): (a) CHL_{clim}; (b) CHL_{inter}. The contour interval is 0.25 °C. The figure is created by the authors using the Grid Analysis and Display System (GrADS) which is available at http://www.iges.org/grads/grads.html.

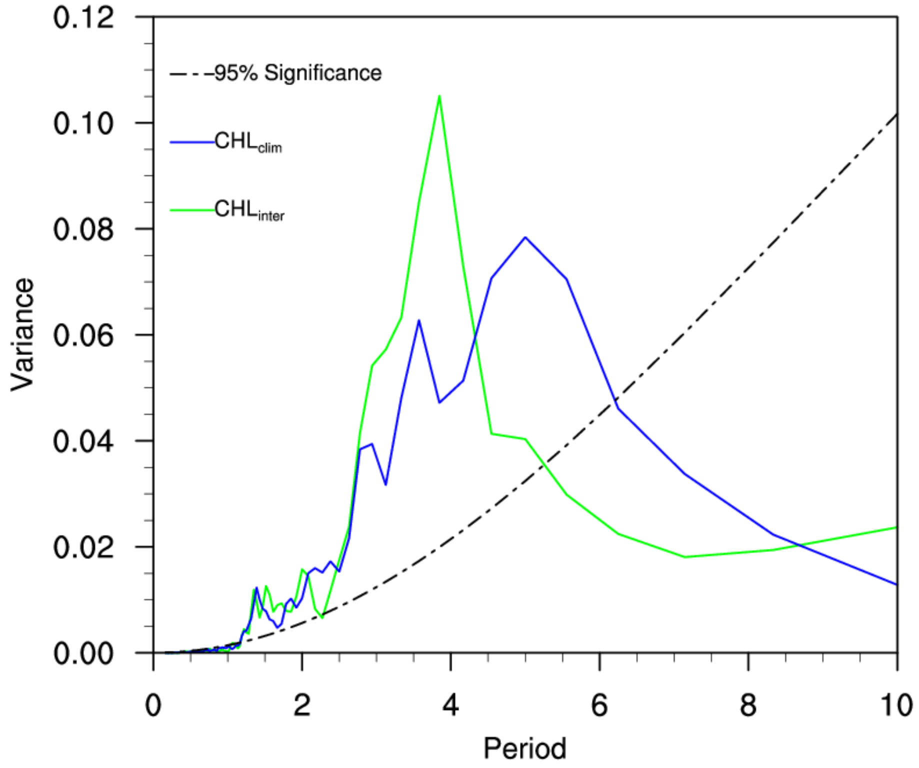

Fig. s14 The power spectra of the Niño3 SST anomalies from long-term runs (150-300 years): CHL_{clim} (blue) run, the CHL_{inter} run (green). The dot-dashed line is the 95% significance level for these runs, assuming a white noise process.

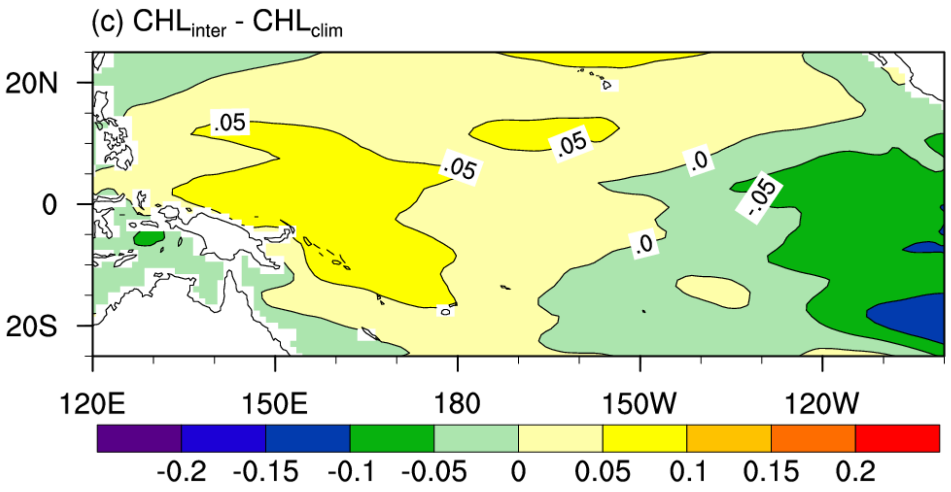

Fig. s15 The difference in the annual-mean SSTs over the tropical Pacific between the CHL$_{clim}$ run and the long-term run (150-300 years). The contour interval is 0.05 °C. The figure is created by the authors using the Grid Analysis and Display System (GrADS) which is available at http://www.iges.org/grads/grads.html.

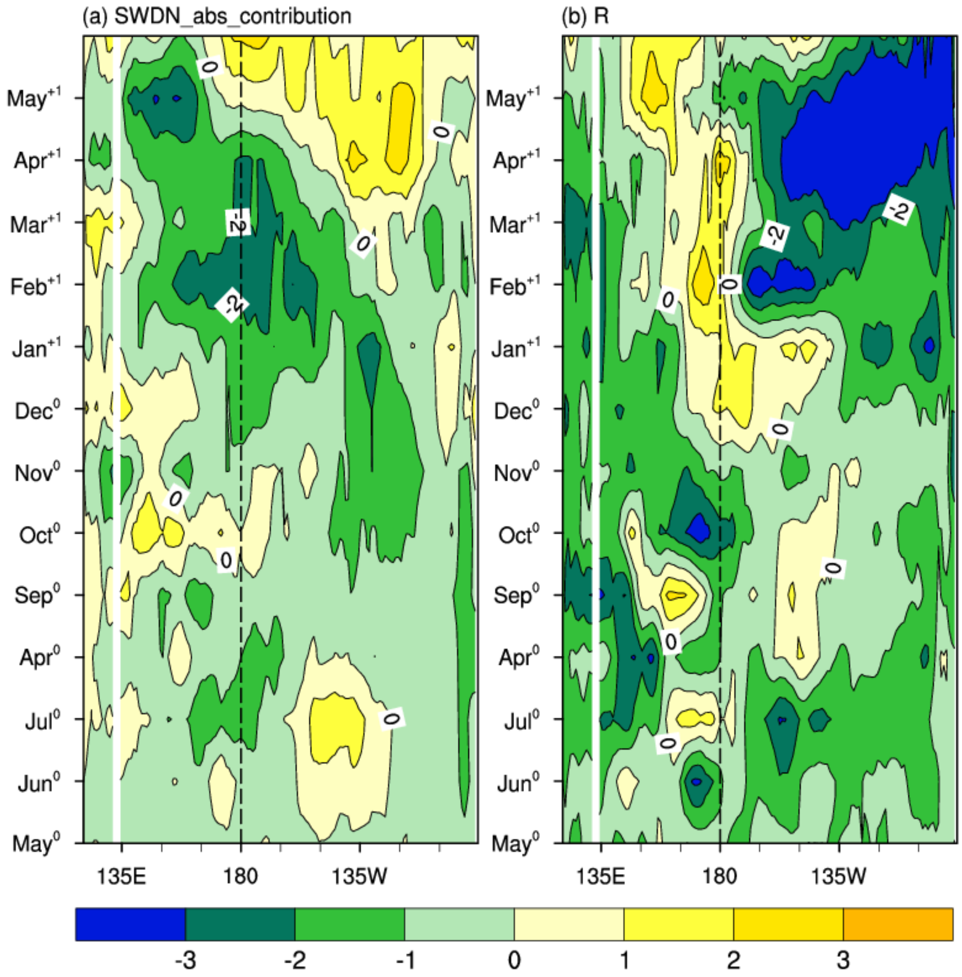

Fig. s16 Longitude-time sections along the equator for the differences between the CHL_{inter}-EN and CHL_{clim}-EN in (a) the contributions of the absorbed SWDN ($\frac{Q_{abs}}{\rho C_p h}$) to the temperature tendency of the first model layer and (b) the effect of vertical mixing (R) on the temperature tendency of the first model layer. The contour interval is 1.0×10^{-6} °C/s.
